# Celecoxib in oncology: targeting the COX-2/PGE_2_ axis to reprogram the tumor immune microenvironment and enhance multimodal therapy

**DOI:** 10.3389/fphar.2025.1691392

**Published:** 2025-11-26

**Authors:** Boyang Kuang, Kuan Yang, Xiaoting Zhong, Yingwei Tan, Yi Zhou, Jianming Ye

**Affiliations:** 1 Department of Oncology, The First Affiliated Hospital, Gannan Medical University, Ganzhou, China; 2 The First Clinical Medical College, Gannan Medical University, Ganzhou, China; 3 Jiangxi Clinical Medical Research Center for Cancer, Ganzhou, China; 4 Department of Oncology, The First Affiliated Hospital, Jinan University, Guangzhou, China

**Keywords:** celecoxib, COX-2 inhibition, tumor microenvironment, combination therapy, precision oncology

## Abstract

Celecoxib, a selective cyclooxygenase-2 (COX-2) inhibitor, has emerged as a multifaceted therapeutic agent in oncology due to its dual anti-inflammatory and antitumor properties. This review synthesizes recent advances in understanding the molecular mechanisms and clinical applications of celecoxib in cancer treatment. Celecoxib not only hinders the proliferation and metastasis of tumor cells by inhibiting COX-2 synthesis, but also inhibits the intratumoral infiltration of regulatory T cells (Tregs) and myeloid-derived suppressor cells (MDSCs) and activates cytotoxic T cells, thereby reshaping the inhibitory immune microenvironment. Preclinical and clinical studies demonstrate its synergistic effects with chemotherapy, radiotherapy, and immunotherapy, particularly in augmenting immune checkpoint blockade efficacy. Despite the breakthrough of celecoxib in the field of oncology treatment, large-scale trials are warranted to validate its long-term safety and biomarker-driven accuracy. This work underscores the potential of celecoxib as a cornerstone in multimodal cancer therapy and provides a roadmap for its integration into personalized treatment paradigms.

## 1 Introduction

Malignant neoplasms are a major global public health challenge, with nearly 20 million new cancer cases as well as 9.7 million cancer deaths in 2022 according to global cancer statistics (Bray et al., 2024), and a complex challenge for clinical oncology, characterized by heterogeneity and the ability to evade immune surveillance. The pathogenesis of cancer involves a variety of factors, including genetic mutations, epigenetic modifications, and the tumor microenvironment, which work together to contribute to tumor growth, progression, and metastasis (Swanton et al., 2024). Among them, the chronic inflammatory state in the tumor microenvironment is a key factor in the occurrence and progression of malignant tumors (Balkwill and Mantovani, 2001; Greten and Grivennikov, 2019). Inflammatory mediators can not only induce angiogenesis and epithelial interstitial and accelerate tumor invasion and metastasis, but also lead to chemoresistance and immunotherapy resistance by reshaping the tumor immune microenvironment. The activation of tumor-associated inflammation is closely related to the activation of the COX-2 pathway, which is highly expressed in a variety of solid tumors. It can drive tumor cell proliferation, angiogenesis, and immune evasion, and is resistant to anti-tumor therapy (Bell et al., 2022; Bell and Zelenay, 2022; Cheki et al., 2018). The selective COX-2 inhibitor celecoxib has attracted much attention due to its precise regulation of inflammatory pathways.

**FIGURE 1:**
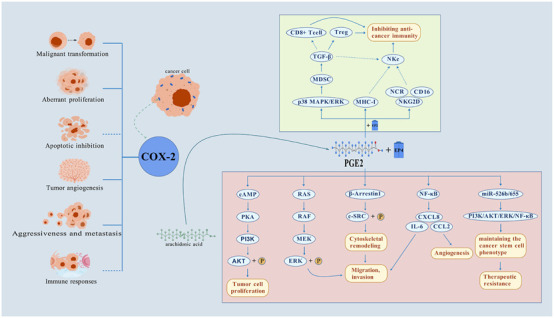
Multiple mechanisms of action of COX-2/PGE_2__2_ signaling axis in tumorigenesis and development. This figure summarizes the core pathway of COX-2 to drive tumor malignancy by catalyzing PGE_2_ synthesis: 1. Activation of pro-cancer signaling: PGE_2_ binds to EP4 receptor and activates multiple pathways such as cAMP/PKA, RAS/MAPK, PI3K/AKT and NF-κB, which synergistically promote tumor cell proliferation, apoptosis inhibition, invasion and metastasis, and angiogenesis. 2. Immune microenvironment remodeling: PGE_2_ binds to EP2 and EP4 receptors to induce the expression of Treg and MDSC inhibitory immune cells and inhibit NK cytotoxicity, weakening the anti-tumor immune response. 3. Stem cell phenotypic maintenance: PGE_2_ maintains cancer stem cell (CSC) properties by stimulating the expression of miR-526 b and miR-655 and mediates treatment resistance. (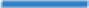 Promotion, 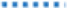 Inhibition).

COX-2 is an enzyme that plays a key role in the inflammatory process and is also implicated in various stages of tumorigenesis. COX-2 is elevated in many types of malignancies (Tołoczko-Iwaniuk et al., 2019), and it has long been found to promote tumor development by modulating malignant transformation, aberrant proliferation, inhibition of programmed apoptosis, tumor angiogenesis, aggressiveness and metastasis, and immune responses (Hashemi Goradel et al., 2019; Trifan and Hla, 2003). Prostaglandin E_2_ (PGE_2_), a type of eicosanoid, is a pro-inflammatory agent that activates various pro-cancer signaling pathways, including cAMP/PKA, ERK, and NF-κB, by binding to EP4 receptors. This binding drives tumor cell proliferation, migration, invasion, and immune evasion. Additionally, PGE_2_can foster immunosuppression and treatment resistance within the tumor microenvironment by suppressing NK cell function and inducing the expression of MDSCs and cancer stem cell (CSC) phenotypes (Wang and Dubois, 2010; Holt et al., 2012; Ching et al., 2020; Mao et al., 2014); COX-2 can facilitate the progression of tumor cells by promoting the transformation of arachidonic acid into PGE_2_, while suppressing anti-cancer immunity (Zelenay et al., 2015; Lira et al., 2024; Li et al., 2020). In summary, COX-2 expression is associated with increased tumor aggressiveness and poor prognosis, highlighting its potential as a therapeutic target for cancer. Celecoxib exerts its antitumor effects by inhibiting COX-2, not only reducing the production of pro-inflammatory prostaglandins, but also influencing various signaling pathways related to tumor growth through its influence. Figure 1 illustrates the multiple mechanisms of action of the COX-2/PGE_2__2_ signaling axis in tumorigenesis and progression.

Celecoxib, which is known as a COX-2 inhibitor selectively, is becoming important in the field of pharmacology due to its unique mechanism that is different from traditional nonsteroidal anti-inflammatory drugs (NSAIDs) (Davies et al., 2000). The typical non-selective NSAIDs, they block both COX-1 and COX-2 enzymes, while celecoxib selectively targets COX-2, which is general upregulated in inflammatory conditions and various malignant tumors (Tołoczko-Iwaniuk et al., 2019). This selectivity reduces the gastrointestinal side effects typically associated with non-selective NSAIDs, making celecoxib a promising candidate for cancer treatment (Rao and Reddy, 2004) (Quiñones and Pierre, 2019). The pharmacological characteristics of celecoxib has been extensively studied, revealing its potential to inhibit proliferation and promote apoptosis of various cancers, including colorectal cancer, liver cancer, breast cancer, and so on (Wen et al., 2020).

The dual effects of anti-inflammatory and potentially anti-cancer properties are important manifestations of celecoxib in cancer research. Numerous studies have shown that it is effective in inhibiting COX-2 expression in tumor cells, thereby mitigating tumor growth and increasing the effectiveness of conventional cancer therapy (Tai et al., 2019). More and more studies have shown that there is a complex interaction network between tumor inflammatory state and tumor progression, so the in-depth analysis of the regulatory mechanism of celecoxib on tumor inflammation by anti-inflammatory drugs will provide a key theoretical basis for realizing tumor immune microenvironment reprogramming and breaking through the treatment bottleneck (Negi et al., 2019).

## 2 Clinical applications: from symptom control to multimodal therapy

### 2.1 Pain relief

The analgesic efficacy of celecoxib in patients with malignancies has been extensively studied for its ability to reduce inflammation and pain response by selectively inhibiting COX-2 and decreasing prostaglandin synthesis, and is commonly used for mild to moderate pain caused by cancer, and in a study by Hou et al., the addition of celecoxib was effective in improving pain in patients with carcinomatous neuralgia (Hou et al., 2021). Furthermore, clinical trials have shown that celecoxib can relieve pain caused by radiotherapy (Ghasemi et al., 2018). In addition, the use of celecoxib can reduce the need for opioids in postoperative patients, thereby reducing opioid side effects such as respiratory depression, nausea, constipation, and high dependence rates (Carpenter et al., 2018). In the treatment of metastatic bone cancer, celecoxib can be used in combination with opioids or other analgesics to significantly enhance the analgesic effect, and the combination of non-steroidal anti-inflammatory drugs can also reduce morphine use (Liu et al., 2017). However, a meta-analysis suggests that while celecoxib is effective in the treatment of chronic pain and inflammatory diseases, the associated risks associated with long-term use, such as cardiovascular disease, need to be carefully considered (Ye et al., 2022). Therefore, celecoxib has shown good analgesic effect in pain management of patients with malignancy, especially in relieving chronic pain caused by cancer, reducing opioid demand, and being used in combination with other drugs, but its potential risks should be carefully considered in the long term.

### 2.2 Combination of celecoxib with other treatments

In addition to analgesic effects, the use of celecoxib in combination with other treatments in malignant tumors is also gradually recognized. An important area of focus is its potential role in enhancing the efficacy of cancer treatment. For example, the combination of celecoxib with chemotherapy can significantly prolong the progression-free survival (PFS) and overall survival (OS) of patients (Bąk and Krupa, 2023). In terms of radiation therapy, celecoxib also exhibited synergistic anti-cancer effects (Mitryayeva et al., 2024). In addition, celecoxib has been shown to enhance anti-tumor by enhancing the effects of PD-1 inhibitors immunity (Hu et al., 2022). Although celecoxib has demonstrated potential synergies in combination with chemotherapy, radiotherapy, and immunotherapy, this does not mean that simply adding celecoxib necessarily improves treatment outcomes (Li et al., 2023; Meyerhardt et al., 2021). The actual effect still depends on the combination of multiple factors, so more clinical studies are needed to verify its long-term safety and efficacy.

#### 2.2.1 Chemotherapy

Celecoxib as a selective COX-2 inhibitor has shown promising prospects in combination with chemotherapy. This strategy is supported by evidence that drugs such as cisplatin and 5-fluorouracil have been shown to enhance COX-2 expression in preclinical models such as lung and colorectal cancer, which not only enhances tumor cell survival, invasion, and angiogenesis, but also drives the production of prostaglandin E_2_ to increase inflammation and recruit immunosuppressive cells such as myeloid-derived suppressor cells and regulatory T cells, thereby limiting the efficacy of chemotherapy and immunotherapy (Bell et al., 2022; Bell and Zelenay, 2022). By targeting this resistance axis, celecoxib can enhance the cytotoxicity of conventional chemotherapeutic agents. or example, In an *in vitro* study and tumor bering mouse model using the SGC-7901/DDP cell lines for gastric cancer, cisplatin combined with celecoxib enhanced cisplatin cytotoxicity in a cyclooxygenase-2-dependent manner (Xu et al., 2015; Xu et al., 2016). Similarly, in an *in vitro* study using human skin cancer, celecoxib combined with doxorubicin was found to significantly reduce cell viability by inhibiting the AKT and COX-2 pathways, thereby promoting cell death (Singh, 2018). And in a MATE analysis of advanced non-small cell lung cancer, celecoxib combined with chemotherapy significantly improved overall response and survival (Zhang et al., 2020). In addition, celecoxib during chemotherapy can also reduce side effects caused by chemotherapy, such hand-foot syndrome (HFS), thereby improving patient tolerance (Shayeganmehr et al., 2023). However, although the combination of celecoxib has been shown to improve survival in some cases, it has also been suggested that it may increase the risk of certain adverse effects, such as hematologic toxicity and cardiovascular events (Zhang et al., 2020). Therefore, in clinical application, the potential benefits and risks of celecoxib need to be weighed against the specific situation of the patient.

#### 2.2.2 Radiotherapy

Celecoxib is often used as a sensitizer for radiation therapy. It may reduce adaptive resistance to radiotherapy by inhibiting COX-2-dependent angiogenesis and tumor aggressiveness (Cheki et al., 2018). Studies have found that celecoxib can enhance the sensitivity of radiotherapy to non-small cell lung cancer cells and promote radiotherapy-induced apoptosis, which may be related to celecoxib’s downregulation of the Akt/mTOR signaling pathway (Zhang et al., 2019). Sun et al. also found that celecoxib could enhance radiotherapy-induced apoptosis (Sun et al., 2017). In a study of radiotherapy for squamous cell carcinoma of the head and neck, celecoxib was found to improve the effectiveness of radiotherapy, especially against the effects of angiogenesis (Mitryayeva et al., 2024). In addition, the use of celecoxib may reduce the adverse effects associated with radiotherapy and help relieve the discomfort associated with radiotherapy (Bi et al., 2019). There are also many clinical trials that have confirmed the therapeutic efficacy of celecoxib in radiotherapy (Liao et al., 2005; Xue et al., 2011; Wang et al., 2014). Therefore, the combination of celecoxib with radiotherapy may provide a new strategy for improving radiotherapy efficacy.

#### 2.2.3 Immunotherapy

In immunotherapy, celecoxib also exhibits a synergistic effect. Tumor cells can induce tumor immune escape *via* the COX-2-PGE_2_ pathway (Jin et al., 2023), and the combination of celecoxib with anti-PD-1 monoclonal antibodies is faster than anti-PD-1 alone to induce tumor eradication (Zelenay et al., 2015). Celecoxib in combination with immune checkpoint inhibitors significantly increases tumor-infiltrating T cell activity and improves anti-tumor immune responses (Cao et al., 2023; Pelly et al., 2021). Zhang et al. found that celecoxib in combination with roscovitine significantly enhanced the anti-tumor immune response by eliminating inflammation-related immunosuppression and reversing IFN-γ-mediated immune resistance (Zhang et al., 2022). In breast cancer models, celecoxib has been demonstrated to reprogram the CAF-like cell-mediated immunosuppressive microenvironment, promote the infiltration of cytotoxic T lymphocytes, and inhibit regulatory T cell (Treg) activity, thereby enhancing the efficacy of immunotherapy (Samoudi et al., 2024). In addition, Pan et al. also found that celecoxib derivatives (2,5-dimethylcelecoxib) can inhibit the expression of programmed cell death protein-1 by regulating the tumor microenvironment and upregulate the expression of NK and T cells, providing a reference for combined immunotherapy,but this derivative is independent of the COX-2 signaling pathway (Pan et al., 2023). The mechanism of COX-2-PGE_2_ pathway promoting progression in malignant tumors through immunosuppression has been continuously explored (Pu et al., 2021), providing a new strategy for COX-2 inhibitor combination immunotherapy (Kosaka et al., 2023; Veltman et al., 2010), and the development of some celecoxib derivatives has also provided some mechanism exploration for non-COX-2 dependence (Tan et al., 2021; Sigler et al., 2025), which may be a new path, but the efficacy of celecoxib combined with immunotherapy needs to be verified by more clinical trials.

#### 2.2.4 Other applications

In addition to the above-mentioned applications, there are some other applications of celecoxib that are still being explored. For example, it is also effective in combination with targeted drugs (Lin et al., 2018; Valverde et al., 2017; Tudor et al., 2021; Xiao et al., 2019), and it can also be used in combination with some novel therapies [such as oncolytic virus therapy (Tang et al., 2020), nano delivery system (Bai et al., 2024), immunophotodynamic therapy (An et al., 2024; Ding et al., 2024)] for anti-tumor, which can help improve the research of celecoxib in cancer and provide more personalized treatment strategies.

## 3 Celecoxib’s antitumor mechanism

### 3.1 The apoptotic induction mechanism of celecoxib

**FIGURE 2:**
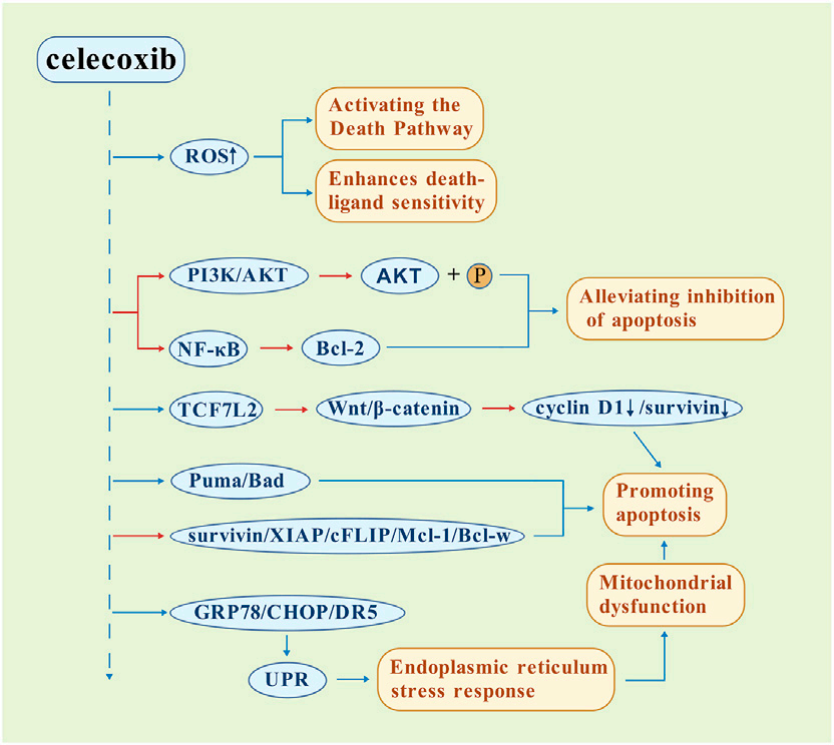
Mechanistic framework of celecoxib-induced apoptosis. The driving role of ROS was revealed, and the inhibition of multiple key survival pathways, the regulation of apoptosis regulatory proteins, and the activation of independent endoplasmic reticulum stress pathways to mitochondrial dysfunction were demonstrated. The Wnt/β-catenin and UPR/ERS pathways are COX-2-independent pathways. (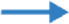
Promotion, 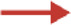 Inhibition).

Celecoxib induces apoptosis through a multi-target mechanism, ranging from upstream signaling regulation to terminal effector activation (Figure 2). The core mechanism initiates with a significant accumulation of reactive oxygen species (ROS), which serve as a pro-apoptotic signaling hub. These ROS not only directly activate downstream death pathways (Sung et al., 2017; Pritchard et al., 2018), but also enhances the sensitivity of cancer cells to death ligands such as FasL/RAIL, demonstrating synergistic pro-apoptotic effects (Zhu et al., 2021). At the regulatory level of key signaling pathways, the drug effectively blocks the anti-apoptotic function of Bcl-2 protein and Akt kinase downstream of NF-kB by inhibiting the activation of NF-kB in the NF-kB pathway and the phosphorylation of Akt kinase in the PI3K/Akt pathway, respectively, and alleviates the inhibitory effect on the cell death program (Li et al., 2020; Hsu et al., 2000). Celecoxib can also inhibit the Wnt/β-catenin pathway by promoting TCF7L2 protein degradation, thereby decreasing the expression of downstream cyclin D1 and survivin and promoting apoptosis, which is a COX-2-independent pathway (Egashira et al., 2017). The molecular regulatory network exhibits a two-way dynamic balance: the upregulation of the expression of pro-apoptotic protein (Puma/Bad) and the downregulation of anti-apoptotic factors (survivin/XIAP/cFLIP/Mcl-1/Bcl-w) create a cascade amplification effect, which significantly promotes the process of apoptosis (Zhu et al., 2021). Additionally, celecoxib upregulates GRP78, C/EBP-homologous protein (CHOP), death receptor 5 (DR5), and activates the endoplasmic reticulum stress response through the unfolded protein response (UPR), opening up another independent apoptosis signaling pathway (Thi Thanh Nguyen and Yoon, 2024). This multi-layered network of action, initiated by oxidative stress, transduced through key pathway nodes (ROS/Akt), and converging on apoptosis executive proteins, explains the unique advantages of celecoxib in combination therapy. This has been confirmed to produce a synergistic sensitization effect when combined with conventional therapy, providing a new direction for optimizing anti-cancer strategies (Qadir et al., 2023).

Notably, substantial evidence underscores the importance of the COX-2-independent pathway in celecoxib-induced apoptosis. For example, celecoxib directly inhibits 3-phosphoinositol-dependent protein kinase-1 (PDK-1), an upstream activator of the survival-promoting Akt pathway. This inhibition attenuates Akt signaling and helps induce apoptosis in cancer cells independent of COX-2 inhibition (Li et al., 2006; Tseng et al., 2006; Kulp et al., 2004). In addition, celecoxib has been shown to inhibit p38 and p55 MAPKs in the JNK pathway and activate pro-apoptotic pathways (Gallicchio et al., 2008). The apoptosis mechanisms of these COX-2-independent pathways offer different ideas for exploration.

### 3.2 The impact of celecoxib on the tumor microenvironment

**FIGURE 3:**
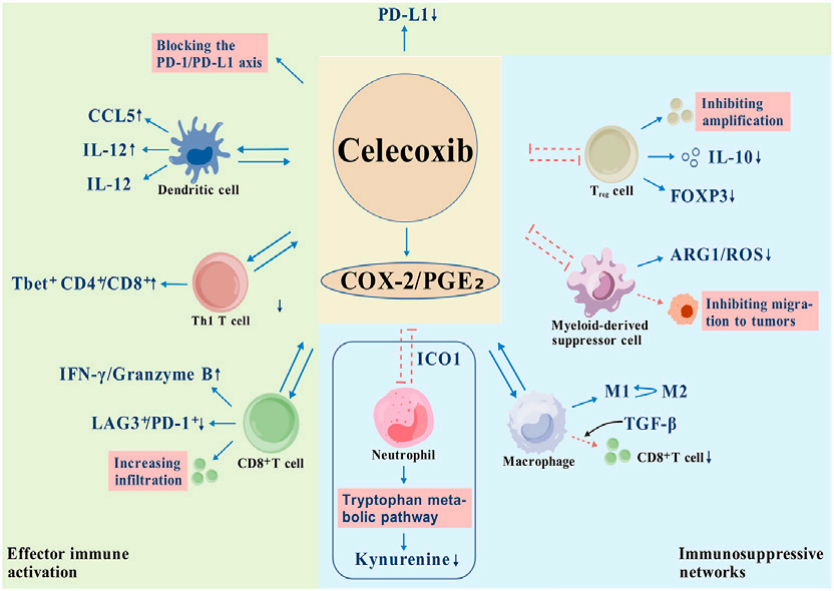
Celecoxib remodels the tumor immune microenvironment by targeting the COX-2/PGE_2_ axis. 1. Immunosuppression is relieved (blue area): ↓Tregs amplification: Immune tolerance is broken by reducing IL-10 secretion and down-regulating FOXP3 expression; ↓MDSCs function: block ARG1/ROS-dependent T cell inhibition and inhibit its migration to tumors; ↓M2 macrophage polarization: reversal of TGF-β-mediated CD8^+^T cell exhaustion; ↓Abnormal tryptophan metabolism: inhibits IDO1-mediated kynurenine accumulation in TANs. 2. Effector immune activation (green area): ↑DC: promotes the production of CCL5 by NK cells, regulates the increase of Th1 cytokine IL-12 and decreases the Th2 cytokine IL-10; ↓Immune checkpoints: block the PD-1/PD-L1 axis and relieve T cell inhibition; ↑CD8^+^T cell function: promotes tumor infiltration, upregulates IFN-γ/granzyme B, and reduces depletion markers (LAG3^+^/PD-1^+^); ↑Th1 immune response: regulates the balance of T cell subsets and forms anti-tumor cell communities. Metabolic-signaling synergistic regulation (box area): Key metabolic interventions: inhibition of IDO1 activity→blocking of tryptophan-kynurenine aberrant metabolism → reduction of immunosuppressive metabolite accumulation. (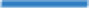 Promotion, 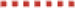
> Inhibition).

Celecoxib is a highly selective COX-2 inhibitor with numerous mechanisms of action within the tumor microenvironment (TME). Its core mechanism is reflected in the bidirectional regulation of the immunosuppressive network and the activation of immune responses (Figure 3) (Jahani et al., 2023; Xun et al., 2021; Cecil et al., 2022; Rao, 2022; Kobayashi et al., 2020; Ouyang et al., 2024; Raaijmakers et al., 2022; Zhang et al., 2013; Qin et al., 2022). First, celecoxib directly inhibits the recruitment and function of immunosuppressive cells by reducing the PGE_2_ level in TME: (1) it decreases the expansion of regulatory T cells (Tregs) and their IL-10 secretion, and downregulates their FOXP3 expression to break immune tolerance (Jahani et al., 2023; Cecil et al., 2022; Kobayashi et al., 2020); (2) it blocks the ARG1/ROS-dependent T cell inhibitory function of myeloid-derived suppressor cells (MDSCs), inhibiting the number and function of MDSCs while inhibiting their migration to tumor tissue (Veltman et al., 2010; Jahani et al., 2023; Ouyang et al., 2024); and (3) it reverses macrophage to M2 phenotypic polarization and reduces TGF-β-mediated CD8+ T cell exhaustion (Xun et al., 2021). Additionally, the drug alleviates the inhibition of T cell activity by interfering with the COX-2/PGE_2_/IDO1 axis and inhibiting abnormal tryptophan metabolism in tumor-associated neutrophils (TANs) (Ouyang et al., 2024). Collectively, hese effects weaken the immunosuppressive barrier within the TME.

At the effector immune cell level, celecoxib enhances the anti-tumor immune response through dual regulation of metabolism and signaling. On one hand, the reduction of PGE_2_ significantly downregulates the PD-L1 expression of tumor cells and myeloid cells, thereby blocking the inhibitory effect of PD-1/PD-L1 immune checkpoints on T cells (Cecil et al., 2022; Rao, 2022). On the other hand, the drug promotes CD8+ T cell infiltration and enhances its function: it improves cytotoxicity by upregulating IFN-γ and granzyme B secretion, while decreasing LAG3^+^/PD-1^+^ depletion marker expression (Rao, 2022; Kobayashi et al., 2020; Raaijmakers et al., 2022). Notably, celecoxib increases the proportion of Th1 T cells (Tbet^+^ CD4^+^/CD8^+^) with antitumor activity by regulating T cell subset homeostasis and forming a cell community conducive to immune attack (Cecil et al., 2022; Rao, 2022). Additionally, dendritic cells (DCs) are essential for the activation of cytotoxic T lymphocytes, and the drug can increase the recruitment of DCs and enhance the ability of DCs: celecoxib can support the recruitment of DCs by promoting the chemokine CCL5 produced by NK cells, and can enhance the DC effect by increasing the Th1 cytokine IL-12 and decreasing the Th2 cytokine IL-10 (Raaijmakers et al., 2022; Zhang et al., 2013; Qin et al., 2022). This dynamic regulation from inhibition release to effect activation reflects its multi-level intervention on the immune microenvironment.

The antitumor mechanism of celecoxib is also involved in key processes of immunometabolic reprogramming. It reverses T cell functional exhaustion by inhibiting indoleamine 2,3-dioxygenase 1 (IDO1) activity, blocking the aberrant metabolism of tryptophan to kynurenine and reducing the accumulation of immunosuppressive metabolites (Ouyang et al., 2024). The synergistic effect of these metabolic interventions with immune signaling pathways further strengthens the position of the COX-2/PGE_2_ axis as a core hub for the regulation of the tumor immune microenvironment.

## 4 Research progress and therapeutic application of celecoxib in a variety of malignant tumors

A growing number of preclinical trials and clinical trials have shown that celecoxib has an important role in the treatment of different malignancies, and it exerts anti-cancer effects through different mechanisms. The following is a summary of the research progress and therapeutic application of celecoxib in different malignant tumors, which is summarized in Table 1. Table 2 shows some clinical trials of celecoxib in different malignancies.

**TABLE 1:** Research progress and therapeutic application of celecoxib in a variety of malignant tumors.

Tumor type	Target/Pathway	Core mechanism of action	Key results	Options	References
Colorectal cancer	COX-2, ROS, Bcl-2	Increasing oxidative stress, down-regulating anti-apoptotic protein	Inhibiting tumor properties, promoting apoptosis of cancer cells	Monotherapy or combination therapy	Xu et al. (2023), Mohammadi et al. (2016), Maniewska and Jeżewska (2021)
Colorectal cancer	PDK-1/Akt, SERCA, Mcl-1, Bcl-2	Inducing tumor cell apoptosis, inhibiting proliferation	Inducing apoptosis in COX-2-negative colon cancer cells	Monotherapy or combination chemotherapy	Jendrossek (2013)
Breast cancer	COX-2/VEGF	Reshaping the tumor immune microenvironment	Inhibiting tumor angiogenesis and cancer cell metastasis	Combination immunotherapy	Bai et al. (2024)
Breast cancer	COX-2/PD-L1	Reshaping the tumor immune microenvironment	Enhancing the killing effect of T lymphocytes, promoting the infiltration of CD8^+^ T cells into tumor tissues	Combination immunotherapy	Bai et al. (2024)
Breast cancer	Bcl-2/Bax/Caspase-3	Upregulating pro-apoptotic genes, downregulating anti-apoptotic genes	Inducing apoptosis in cancer cells	Combination chemotherapy	Bardaweel et al. (2022), Hedayat et al. (2023)
Breast cancer	COX-2/PGE_2_	Reducing PGE_2_ synthesis, promoting DC maturation and T cell activation	Enhancing immunogenicity cell death (ICD)	Combination chemotherapy	Qian et al. (2024)
Lung cancer	JNK/PI3K, ULBP-1	Upregulation of ULBP-1 expression enhances NKc cytotoxicity	Enhancing NKc cell-mediated tumor cell lysis	Monotherapy	Kim et al. (2020)
Lung cancer	EGFR/PI3K/AKT, EGFR/ERK/AKT	Affecting the cell cycle, promoting radiation-induced apoptosis	Combination of targeted drugs to enhance radiotherapy sensitivity	Combination of targeted therapy and radiotherapy	Sun et al. (2017), Zhang et al. (2021)
Lung cancer	Akt/mTOR, Ido1/ER	Inhibiting Ido1 expression and enhances ROS/endoplasmic reticulum stress	Enhancing immunogenicity cell death (ICD)	Combination radiotherapy	Qadir et al. (2023), Zhu et al. (2024)
Lung cancer	COX2/PGE_2_	Blocking immune escape after STING activation	Controling tumor growth, reducing recurrence	Combined with STING agonists	Lemos et al. (2020)
Prostate cancer	AKT, EGFR/ErbB, hnRNP K, NF-κB	Multi-signaling pathway regulation	Inducing castration-resistant (CRPC) apoptosis, inhibiting the invasive phenotype	Monotherapy or combination targeted therapy	Benelli et al. (2019)
Prostate cancer	COX-2, Glut-1, TrxR, Prx-6	Glut-1 inhibition and oxidative stress induction	Synergistically inhibiting the proliferation of cancer cells, reducing glutathione and increasing ROS	Nanoliposome	Tian et al. (2019)
Prostate cancer	COX-2/PGE_2_	COX-2 pathway inhibition	Improving progression-free survival (PFS)	Combined androgen deprivation therapy (ADT)	Landre et al. (2019)
Prostate cancer	COX-2	Inhibiting tumor properties and reducing radiotherapy resistance	Enhancing radiation sensitivity, reducing the rate of tumor recurrence	Combination radiotherapy	King et al. (2020)
Head and neck cancer	ROS/JNK	Activation of the ROS/JNK axis	Inducing apoptosis and inhibiting cancer cell proliferation	Derivative monotherapy	Tan et al. (2021)
Head and neck cancer	PGE_2_/ANGPTL4/MMP1	COX-2 activity inhibition, ANGPTL4 expression blockade	Reducing tumor invasion, endothelial cell adhesion, and cancer cell metastasis	Monotherapy	Chiang et al. (2020)
Head and neck cancer	COX-2/MDSCs	Inhibition of MDSCs *via* COX-2-mediated immunosuppression	Targeting MDSCs to regulate the tumor microenvironment	Novel mucoadhesive cube sponge	Mabrouk et al. (2023)
Head and neck cancer	EMT markers, ALDH	Inhibition of EMT and stem cell properties	Reducing tumor aggressiveness and stem cell properties	Novel mucoadhesive cube sponge	Mabrouk et al. (2023)
Hepatocellular carcinoma	PNO1, AKT/mTOR	PNO1 expresses inhibition, AKT/mTOR signaling pathway blockade	Inhibiting tumor growth and metastasis	Monotherapy	Dai et al. (2019)
Pancreatic cancer	Neu-1	Inhibition of Neu-1 activity, inhibition of EGFR dimerization and phosphorylation	Inducing apoptosis in cancer cells	Monotherapy	Qorri et al. (2020)
Cervical cancer	OXPHOS, PINK-1/Parkin, Glycoprotein-P	Inducing mitophagy and ROS↑, and inhibit glycoprotein-P activity	Inhibiting cancer cell proliferation, promoting apoptosis, reversing chemotherapy resistance	Combination chemotherapy	Robledo-Cadena et al. (2024)
Gastric cancer	Caspase-3/8/9, p53	Caspase cascade activation, apoptosis induction	Increasing efficacy, reducing side effects	Combination chemotherapy	Badalanloo et al. (2022)
Glioblastoma	ETC	Inhibiting cell death through the mitochondrial metabolic pathway	Reversing chemotherapy resistance	Combination chemotherapy	Yin et al. (2021)
Glioblastoma	NF-κB	Inhibiting NF-κB expression	Inhibit GBM proliferation and increase efficacy	Combination chemotherapy	Ahsan et al. (2023)

Abbreviations: DC, dendritic cell, NKc, natural killer cell, ULBP-1 UL16-binding protein 1, IDO1 indoleamine 2,3-dioxygenase 1, ROS, reactive oxygen species, STING, stimulator of interferon genes, Glut-1 glucose transporter-1, ANGPTL4 angiopoietin-like 4, MDSCs, myeloid-derived suppressor cells, EMT, epithelial-mesenchymal transition, JNK c-Jun N-terminal kinase, PNO1 RNA, binding gene partner of NOB1, mTOR, mammalian target of rapamycin, Neu-1 neuraminidase-1, ETC, electron transport chain.

**TABLE 2:** Clinical trials of celecoxib in different malignancies.

Trial ID	Cancer type	Celecoxib use	Combination	Phase	Outcome	References
NCT03926338	Colorectal cancer	200 mg BID	Toripalimab	II	High complete response rate and safety	Mostafa et al. (2022)
NCT03645187	Colorectal cancer	200 mg BID	FOLFIRI	Randomized	↑ ORR	Meyerhardt et al. (2021)
NCT01150045	Colorectal cancer	400 mg QD	FOLFOX	Randomized	3-year DFS no improvement (76.3% vs. 73.4%)	Gandhi et al. (2024)
NCT04081389	Breast cancer	200 mg BID	Paclitaxel	I	Safety, improve TME	Coombes et al. (2021)
NCT02429427	Breast cancer	400 mg QD	Single	Randomized	5-year DFS (84% vs. 83%)	Bayraktar et al. (2020)
NA	Breast cancer	400 mg BID	Single	II Pilot study	Well-tolerated, ↑ IGFBP-1, ↓ IGFBP-3	Edelman et al. (2017)
NCT01041781	Lung cancer	400 mg BID	Standard chemotherapy	III	No survival difference	Nakai et al. (2020)
UMIN000003649	Prostate cancer	200 mg QD	Radiotherapy and tamsulosin	Randomized	↑ 5-year biochemical recurrence-free rate (98.5% vs.93.4%)	Fontana et al. (2009)
EudraCT 2005-005967-27	Prostate cancer	200 mg BID	CTX	Randomized	MPFS (3 m), OS (21 m)	Patil et al. (2020)
CTRI/2015/11/006,388	Head and neck cancer	200 mg BID	MCT (Methotrexate)	III	↑ OS (7.5 m vs. 6.1 m), ↓ adverse effect (19% vs. 30%)	Patil et al. (2020)
CTRI/2015/11/006,388	Head and neck cancer	200 mg BID	MCT (Methotrexate)	III	↑ OS (7.5 m vs. 6.1 m), ↓ adverse effect (19% vs. 30%)	Patil et al. (2023)
CTRI/2020/11/028,953	Head and neck cancer	200 mg BID	TMC combined with nivolumab	Randomized	↑ OS (6.7 m vs. 10.1 m)	Kapoor et al. (2025)
CTRI/2021/09/036,296	Head and neck cancer	200 mg BID	Triple OMCT	III	↑ OS (5.0 m vs. 3.1 m)	Kim et al. (2025)
NCT00400374	Head and neck cancer	400 mg BID	Erlotinib	I/II	Safety, excellent SPT-free survival	Lipton et al. (2010)
NA	Pancreatic cancer	400 mg BID	Gemcitabine + irinotecan	II	MOS (18 m), ↑QOL	Allegrini et al. (2012)
EudraCT 2007-000065-38	Gastric cancer	200 mg BID	UFT and CTX	II	PFS (2.7 m), OS (7.1 m)	O'Rawe et al. (2022)
ACTRN12619001078145	Glioblastoma	—	RAS modulators	I	↑ MOS (19.9 vs. 14.6 m)	Kamali et al. (2020)
IRCT20171225038070N1	Bbladder cancer	100 mg BID	Intravesical BCG therapy	Randomized	↓ adverse effect (dysuria)	Noronha et al. (2022)
CTRI/2015/09/006,204	Esophageal carcinoma	200 mg BID	Methotrexate (after CRT)	II	PFS no improvement	Mostafa et al. (2022)

ORR, objective response rate; DFS, disease-free survival; TME, tumor microenvironment; IGFBP, insulin-like growth factor-binding protein; OS, overall survival; MCT, metronomic chemotherapy; TMC, triple metronomic chemotherapy; OMCT, oral MCT; SPT, secondary primary tumors; QOL, quality of life; CTX, cyclophosphamide; UFT, tegafur; RAS, renin-angiotensin system; CRT, chemoradiotherapy; PFS, progression-free survival; BCG, *Bacillus* Calmette-Guérin.

### 4.1 Colorectal cancer

Celecoxib has proven to be an effective selective COX-2 inhibitor in the treatment of colorectal cancer (CRC). Research indicates that the drug augments the anti-tumor effects of various medications, including neoadjuvant therapies, by influencing mechanisms such as cell cycle regulation and apoptosis pathways. Studies by Xu and Mohammadi et al. using human colorectal cancer cell lines HCT116, HT-29, and nude mouse models have shown that celecoxib can inhibit the biological behavior of colorectal cancer cells, change the cell cycle, induce apoptosis, and enhance the anti-tumor efficacy of the drug when combined with other drugs [5-(4-hydroxyphenyl)-3H-1,2-dithiole-3-thione (ADT-OH), heat shock protein 90 (HSP90)] (Xu et al., 2023; Mohammadi et al., 2016). Additionally, the results of randomized, placebo-controlled, double-blind clinical studies of several NSAIDs found that celecoxib helps chemoprevention of colorectal cancer and may help reduce the incidence of CRC in high-risk populations (Maniewska and Jeżewska, 2021). Furthermore, the drug’s capacity to target inflammatory pathways that are linked to tumor progression makes it a valuable addition to CRC treatment strategies (Jendrossek, 2013), potentially resulting in increased disease-free survival rates for patients (Hu et al., 2023).

### 4.2 Breast cancer

In the context of breast cancer, celecoxib has been investigated for its potential impact on the efficacy of standard treatments (Bardaweel et al., 2022), particularly in the latest research concerning triple-negative breast cance (Bai et al., 2024). Hedayat et al. demonstrated that paclitaxel in combination of paclitaxel with celecoxib can significantly reduce the viability of breast cancer cells (Hedayat et al., 2023), and it can enhance the efficacy of paclitaxel in inducing immunogenic cell death in tumor cells (Qian et al., 2024). However, the effects of celecoxib in breast cancer may vary, depending on COX-2 expression and estrogen receptor (ER) status, highlighting the necessity for an individualized approach to therapy (Hamy et al., 2019). In an experimental study, celecoxib was found to exhibit different pro-apoptotic effects across different breast cancer subtypes (Wang et al., 2017). These findings emphasize the importance of further exploring the role of celecoxib in the management of breast cancer, particularly in optimizing treatment options for specific patient populations.

### 4.3 Lung cancer

Lung cancer, with the highest incidence and mortality rates worldwide, is the subject of ongoing research into celecoxib, particularly for its potential to modulate the tumor microenvironment and improve the effectiveness of synergistic treatments. Its ability to downregulate COX-2 expression contributes to increased susceptibility to natural killer cell cytotoxicity (Kim et al., 2020), and it can also serve as a radiosensitizer for lung cancer (Zhang et al., 2019; Sun et al., 2017; Zhang et al., 2021), underscoring its potential as a therapeutic adjunct. There is evidence that targeted drugs in combination with celecoxib can enhance efficacy by modulating apoptosis (Zhang et al., 2021; Qadir et al., 2023). Zhu et al. discovered that celecoxib can influence the immune response in lung cancer patients and may improve prognosis when combined with ICD inducer (Zhu et al., 2024). Furthermore, the combination of celecoxib can boost antitumor response and overcome resistance to lung cancer STING agonist treatment (Lemos et al., 2020). Nonetheless, due to variations in patient responses, further exploration is necessary to ascertain the benefits of celecoxib in treating lung cancer.

### 4.4 Prostate cancer

Celecoxib has emerged as a significant contender in the realm of prostate cancer therapy, particularly in managing castration-resistant disease. It modulates pathways associated with castration resistance (CRPC) progression, curbing cell growth and prompting apoptosis *via* AKT inhibition, PARP-1 cleavage, and the proteasomal degradation of the anti-apoptotic protein Mcl-1 (Benelli et al., 2019). A study by Tian et al. revealed that celecoxib suppresses tumor growth and metastasis by targeting pathways integral to androgen receptor signaling and inflammation (Tian et al., 2019). Clinical trials have also demonstrated that combining celecoxib with other drugs, such as docetaxel, leads to enhanced treatment efficacy and improved quality of life (Landre et al., 2019). In a retrospective study, the drug’s capacity to modulate PSA levels in radiotherapy patients underscored its potential in controlling the progression of prostate cancer (King et al., 2020). As ongoing research continues to unravel the intricate interactions of celecoxib within prostate cancer biology, integrating it into standard treatment protocols may offer new strategies to enhance outcomes for patients afflicted with this complex malignancy.

### 4.5 Head and neck cancer

The role of celecoxib in head and neck cancer (HNC) has been extensively studied, and it has demonstrated significant anti-cancer potential due to its anti-inflammatory properties and its ability to modulate the tumor microenvironment. Celecoxib inhibits the COX-2/PGE_2_ signaling pathway, reduces vascular endothelial growth factor (VEGF) expression, and suppresses the proliferation, growth, and metastasis of head and neck cancer cells, while also decreasing PGE_2_-mediated immune escape (Chiang et al., 2020). Mabrouk et al. also observed that the growth and spread of oral squamous cell carcinoma (OSCC) can be retarded by inhibiting tumor-associated inflammatory factors (e.g., COX-2, IL-6, TGF-β), and by modulating the function of myeloid-derived suppressor cells (MDSCs), which helps to attenuate the immune escape mechanism of tumors. Their cube sponge system for celecoxib administration is a promising approach (Mabrouk et al., 2023). And in a recent *in vitro* study using human HNC cell lines, celecoxib was found to exert anti-cancer effects on PIK3CA-mutated head and neck cancer cells through endoplasmic reticulum stress, reactive oxygen species, and mitochondrial dysfunction (Thi Thanh Nguyen and Yoon, 2024). While celecoxib exerts its anticancer effects in this cancer type through multiple mechanisms, large-scale clinical data are still required to substantiate the efficacy and safety of its clinical application.

### 4.6 Other types of tumors

In addition to colorectal cancer, breast cancer, lung cancer, prostate cancer, and head and neck cancer, the application of celecoxib in other types of malignant tumors has gradually attracted attention. For instance, some *in vitro* studies have shown that celecoxib may inhibit the growth of hepatocellular carcinoma by targeting PNO1 (Dai et al., 2019), and it can induce apoptosis in pancreatic cancer cells by targeting mammalian neuraminidase-1 (Qorri et al., 2020). Regarding cervical cancer, the combination of celecoxib with conventional chemotherapy exhibits synergistic effects, hindering tumor progression *via* multiple mechanisms (Robledo-Cadena et al., 2024). In the context of gastric cancer, celecoxib itself exerts cytotoxic effects on cancer cells; notably, its combination with topotecan significantly enhances therapeutic efficacy (Badalanloo et al., 2022). Moreover, extensive *in vitro* and *in vivo* studies reveal that it not only effectively inhibits glioblastoma (GBM) cell proliferation but also potentiates the efficacy of temozolomide against chemotherapy resistance (Yin et al., 2021; Ahsan et al., 2023; Pak et al., 2025), while concurrently acting as a radiosensitizer for radiation-resistant CD133 (+) GBM cells (Ma et al., 2011). Notably, a novel celecoxib derivative can cross the blood-brain barrier (BBB) to inhibit recurrence of brain malignancies in animal models (Shen et al., 2025). As research into the effects of celecoxib on malignant tumors continues to expand, it offers new perspectives and avenues for cancer treatment.

## 5 Side effects and safety of celecoxib and other drugs that inhibit the COX-2/PGE_2_ axis

Celecoxib is widely used for its anti-inflammatory and analgesic properties, with a well-defined safety profile. The most significant concerns involve potential gastrointestinal (GI), cardiovascular (CV), and renal adverse effects. Compared to non-selective NSAIDs (e.g., ibuprofen, naproxen), which inhibit both COX-1 and COX-2, celecoxib’s selectivity for COX-2 significantly reduces the risk of GI mucosal injury, with GI event rates as low as 0.34% (Yeomans et al., 2018). However, high doses (>400 mg/day) or long-term use may increase the risk of myocardial infarction and stroke (Obeid et al., 2022). The PRECISION trial further indicated that CV risk correlates with treatment duration and dose (Pepine and Gurbel, 2017). Notably, some cancer-specific studies have not observed a significant increase in CV events (Hu et al., 2023; Coombes et al., 2021). Renal side effects, such as edema and hypertension, are less common but require monitoring (Biase et al., 2024). Despite these potential risks, the incidence of adverse effects associated with celecoxib use in oncology settings, particularly at therapeutic doses and durations relevant to cancer treatment, is generally manageable.

While celecoxib is the most extensively studied selective COX-2 inhibitor in oncology, other pharmacological agents targeting this axis exist. Rofecoxib is also a selective COX-2 inhibitor and has been withdrawn from the market due to cardiovascular safety concerns, which limits its long-term use in cancer prevention (Bresalier et al., 2005). Etoricoxib, another selective COX-2 inhibitor, has shown significant anti-proliferative and pro-apoptotic effects in preclinical studies in lung and hepatocellular carcinoma, highlighting the co-antitumor potential of this drug class in addition to celecoxib (Md et al., 2021; Ali et al., 2022). Moreover, diclofenac is a non-selective nonsteroidal anti-inflammatory drug but has a strong affinity for COX-2, and its anticancer efficacy observed in studies in colorectal cancer and melanoma is related not only to COX-2 inhibition, but also to the induction of oxidative stress and the regulation of oncogenic signaling pathways (Yilmaz et al., 2021; Qin et al., 2025). These comparisons put celecoxib’s position in context: its risk is a category consideration, but its extensive oncological evidence base and unique pharmacokinetic profile support its sustained action. The presence of multiple drugs targeting this axis highlights its therapeutic effectiveness and offers alternatives for future research and potential combination strategies.

## 6 Discussion and future perspectives

Celecoxib has firmly established itself not only as an adjunctive analgesic or anti-inflammatory agent in oncology, but also as an effective immunomodulator capable of reshaping the tumor immune microenvironment (TIME). This review synthesizes compelling evidence for its anti-tumor efficacy, both as monotherapy and in a multimodal regimen, fundamentally stemming from its ability to disrupt the immunosuppressive COX-2/PGE_2_ axis. By inhibiting Tregs, MDSCs, and M2 macrophage polarization while enhancing CD8^+^ T cell infiltration, cytotoxicity, and dendritic cell function, celecoxib effectively eliminates key barriers to anti-tumor immunity. Synergy with chemotherapy (e.g., enhancing drug toxicity and reducing cancer cell viability), radiotherapy (e.g., enhancing radiosensitivity by inhibiting the Akt/mTOR pathway), and especially immunotherapy (e.g., inhibiting the immunosuppressive environment) (Section 2.2), underscores its versatility as a cornerstone of contemporary cancer treatment strategies.

As noted above, this preclinical research evidence consistently demonstrates a clear mechanistic principle across different cancer types, revealing the anti-cancer ability of celecoxib, sensitizing tumors to conventional therapies, and effectively reprogramming the immunosuppressive tumor microenvironment. In the clinical field (Table 2), pivotal trials have successfully translated this commitment into tangible benefits, with multiple studies showing significant improvements in outcomes. However, existing limitations must also be acknowledged. Preclinical models often employ celecoxib concentrations that may not be clinically achievable while elucidating key mechanisms, raising questions about translational relevance (Tołoczko-Iwaniuk et al., 2019). In addition, the heterogeneity of clinical trial outcomes [e.g., the significant efficacy of celecoxib in combination with toripalimab in the phase II PICC trial contrasted with the negative results of the phase III CALGB 30801 trial in patients with unselected NSCLC and the lack of a disease-free survival benefit in the CALGB/SWOG 80702 trial (Hu et al., 2022; Meyerhardt et al., 2021; Edelman et al., 2017)] highlights the limited efficacy of celecoxib and is highly dependent on the context, specifically tumor type, combination agent, and vital patient selection biomarkers. Therefore, while the available data firmly establish celecoxib as a compelling therapeutic-beneficial drug, there is an urgent need for more and more rigorously designed, biomarker-selected clinical trials to definitively determine its role in multimodal oncology.

More notably, the true transformative potential of celecoxib extends beyond its established mechanisms, pointing to new areas of cancer treatment that require attention:

Deepening the Understanding of Immunometabolic Reprogramming: While celecoxib’s immunomodulatory effects are well described, its impact on immunometabolism within TIME represents a critical and underexplored aspect. The ability of celecoxib to inhibit IDO1 activity and correct abnormal tryptophan metabolism in tumor-associated neutrophils (TANs) and other cells directly links COX-2 inhibition to metabolic pathways that control T cell depletion (Ouyang et al., 2024). This makes celecoxib unique at the intersection of inflammation, metabolism, and immunity. Future research should dissect how celecoxib-induced metabolic shifts (e.g., reduced kynurenine accumulation) interact synergistically with immune signaling pathways to create a relaxed environment for effector cells, potentially revealing novel combinatorial targets beyond IDO1 and deepening our understanding of its role in overcoming metabolic immunosuppression.

Patient heterogeneity and towards personalized treatment: The efficacy of celecoxib is heterogeneous among different patient populations, and future clinical translation may depend on identifying patient subgroups most likely to benefit. For example, tumor biology, where COX-2 overexpression may predict benefit, as shown by the trend of improved survival in this subgroup in a negative phase III NSCLC trial, underscores the importance of biomarkers driving patient selection (Edelman et al., 2017). Second, pharmacogenomics, genetic polymorphisms in CYP2C9 (the main metabolizing enzyme of celecoxib) can significantly alter drug clearance, meaning that individuals with adverse metabolizer genotypes may experience higher drug exposure, affecting efficacy and the risk of dose-dependent adverse effects (Dean et al., 2016; Kim et al., 2017). Finally, in terms of gender differences, sex-specific differences in the use of NSAIDs were observed in an epidemiological study of bladder cancer risk, suggesting that sex hormones or other physiological differences may modulate the COX-2/PGE_2_ axis (Daugherty et al., 2011). Therefore, it is crucial to integrate robust biomarkers that include tumor COX-2 status, germline genetic variants, and potentially sex-specific factors into the design of future clinical trials. These parameters precisely define the role of celecoxib in personalized treatment modalities.

Precision Delivery and Biomarker-Driven Integration: The limitations of systemic celecoxib, particularly the cardiovascular risks associated with long-term/high-dose use (Obeid et al., 2022; Pepine and Gurbel, 2017), necessitate more informed dosing strategies and refined patient selection. Innovations in nanodelivery systems offer a very promising solution (Bai et al., 2024). By encapsulating celecoxib or combining it with other drugs (e.g., gemcitabine, immunomodulators) in nanoparticles, these systems enhance tumor-specific targeting, minimize off-target effects (potentially mitigating CV risk), and can achieve localized high-dose effects critical for effective TIME remodeling (Zhang et al., 2024; Liu et al., 2024). In addition, the efficacy of these advanced delivery methods can be evaluated in combination with robust biomarker-guided strategies. In addition to static COX-2 expression assessments, dynamic monitoring utilizing circulating factors (e.g., VEGF reflecting angiogenesis/inflammatory regulation, IL-8) (Passaro et al., 2024; Dovizio et al., 2012; Perroud et al., 2016), single-cell sequencing to analyze dynamic changes in immune cell subsets within the TME (Pan et al., 2023; Zheng et al., 2021), and functional testing using patient-derived 3D organoid models capable of predicting real-time treatment response are key to identifying subgroups of patients most likely to benefit (Xie et al., 2021; Ban et al., 2024). This comprehensive approach shifts the paradigm from a single modality to a truly personalized combination therapy, defining the optimal timing, sequence, and dosage of celecoxib in a complex multimodal protocol.

Expanding the Portfolio Arsenal with Emerging Immunotherapies: Celecoxib could significantly enhance next-generation immunotherapies, an exciting and underexplored avenue. Its potential to enhance the invasion, activation state, and antitumor function of tumor-infiltrating lymphocytes (TILs) through metabolic regulation provides a strong case for its combination with TIL therapy (Kosaka et al., 2023; Cecil et al., 2022; Ferrandina et al., 2006). In immunophotodynamic therapy (IPDT), celecoxib significantly broadens the therapeutic window by stabilizing mitochondrial membrane potentials, thereby reducing phototoxicity in normal tissues and simultaneously enhancing ROS-induced immunogenic cell death (An et al., 2024; Agostinis et al., 2011). Similarly, in oncolytic virutherapy, celecoxib can enhance immune efficacy against glioma by inhibiting the immunosuppressive environment (Tang et al., 2020). In addition, experimental studies have established a link between next-generation immune checkpoints (such as LAG3) and celecoxib (Cecil et al., 2022; Hashemi et al., 2025), and more combination regimens are expected to be used in tumor treatment in the future, although these still require a large amount of research to promote clinical translation.

Harnessing the Potential of Novel Derivatives: The discovery of COX-2-independent antitumor effects of certain celecoxib derivatives opens up a critical avenue. Derivatives such as 2,5-dimethylcelecoxib can induce apoptosis through ROS/JNK activation or modulate PD-1 expression through mechanisms involving the gut microbiota-AMPK-mTOR axis (Pan et al., 2023; Tan et al., 2021), thereby preserving therapeutic benefits while potentially circumventing the cardiovascular risks associated with conventional COX-2 inhibition. More translationally meaningful novel structure-modifying derivatives (designed to cross the BBB) have demonstrated efficacy in inhibiting tumor recurrence in animal models of GBM (Shen et al., 2025). This breakthrough addresses a key challenge in targeting CNS tumors and provides a powerful new tool for modulating the local immunosuppressive microenvironment of GBM. Prioritizing the development and clinical evaluation of these innovative derivatives is a strategic avenue to accelerate the translation of safer and more effective celecoxib therapies.

In conclusion, celecoxib transcends its origins as a mere COX-2 inhibitor. It is a multifaceted immunomodulator capable of reprogramming the immunosuppressive TIME, thereby enhancing the efficacy of multiple anti-cancer modalities. By focusing research on the aforementioned frontier areas–deepening our understanding of immunometabolic crosstalk, advancing the precise delivery of dynamic biomarker guidance, exploring synergies with cutting-edge immunotherapies like TIL and IPDT, and actively developing novel derivatives–celecoxib has the potential to evolve from a valuable adjuvant to a central pillar of the next-generation of immune-focused medicines for multimodal cancer treatment.

## 7 Conclusion

Celecoxib effectively reprograms the immunosuppressive tumor microenvironment by targeting the COX-2/PGE_2_ axis, inhibiting immunosuppressive cells (Tregs, MDSCs, M2 macrophages) while enhancing CD8^+^ T cell infiltration and activity. Its synergistic enhancement of chemotherapy, radiotherapy, and immunotherapy, especially immune checkpoint blockade, stems primarily from disrupting this critical axis, thereby overcoming key mechanisms of treatment resistance and immune evasion. Nanodelivery strategies offer promising avenues for improving tumor targeting and mitigating systemic risks, particularly cardiovascular problems associated with long-term use. Future integration into cancer treatment requires biomarker-driven patient selection and clinical validation. Prioritizing the development of novel derivatives and exploring synergies with next-generation immunotherapies (e.g., TILs, IPDTs) will further unlock their potential as a cornerstone of multimodal precision oncology.

## References

[B1] Agostinis P. Berg K. Cengel K. A. Foster T. H. Girotti A. W. Gollnick S. O. (2011). Photodynamic therapy of cancer: an update. CA Cancer J. Clin. 61 (4), 250–281. 10.3322/caac.20114 21617154 PMC3209659

[B2] Ahsan H. Malik S. I. Shah F. A. El-Serehy H. A. Ullah A. Shah Z. A. (2023). Celecoxib suppresses NF-κB p65 (RelA) and TNFα expression signaling in glioblastoma. J. Clin. Med. 12 (20), 6683. 10.3390/jcm12206683 37892820 PMC10607796

[B3] Ali G. Omar H. Hersi F. Abo-Youssef A. Ahmed O. Mohamed W. (2022). The protective role of etoricoxib against diethylnitrosamine/2-acetylaminofluorene- induced hepatocarcinogenesis in Wistar rats: the impact of NF-κB/COX-2/PGE2 signaling. Curr. Mol. Pharmacol. 15 (1), 252–262. 10.2174/1874467214666210708103752 34238176

[B4] Allegrini G. Di Desidero T. Barletta M. T. Fioravanti A. Orlandi P. Canu B. (2012). Clinical, pharmacokinetic and pharmacodynamic evaluations of metronomic UFT and cyclophosphamide plus celecoxib in patients with advanced refractory gastrointestinal cancers. Angiogenesis 15 (2), 275–286. 10.1007/s10456-012-9260-6 22382585 PMC3338912

[B5] An J. Lv K. P. Chau C. V. Lim J. H. Parida R. Huang X. (2024). Lutetium texaphyrin-Celecoxib conjugate as a potential immuno-photodynamic therapy agent. J. Am. Chem. Soc. 146 (28), 19434–19448. 10.1021/jacs.4c05978 38959476 PMC12005638

[B6] Badalanloo K. Naji T. Ahmadi R. (2022). Cytotoxic and apoptotic effects of celecoxib and topotecan on AGS and HEK 293 cell lines. J. Gastrointest. Cancer 53 (1), 99–104. 10.1007/s12029-020-00434-8 33200341

[B7] Bai L. Liu H. You R. Jiang X. Zhang T. Li Y. (2024). Combination nano-delivery systems remodel the immunosuppressive tumor microenvironment for metastatic triple-negative breast cancer therapy. Mol. Pharm. 21 (5), 2148–2162. 10.1021/acs.molpharmaceut.3c00242 38536949

[B8] Bąk U. Krupa A. (2023). Challenges and opportunities for celecoxib repurposing. Pharm. Res. 40 (10), 2329–2345. 10.1007/s11095-023-03571-4 37552383 PMC10661717

[B9] Balkwill F. Mantovani A. (2001). Inflammation and cancer: back to virchow? Lancet 357 (9255), 539–545. 10.1016/S0140-6736(00)04046-0 11229684

[B10] Ban Q. Y. Li H. S. Jiang X. X. Liu M. Ge X. Y. Lu M. J. (2024). Current applications of colorectal cancer organoids: a review. J. Gastrointestin Liver Dis. 33 (2), 269–277. 10.15403/jgld-5388 38944855

[B11] Bardaweel S. K. Dahabiyeh L. A. Akileh B. M. Shalabi D. D. AlHiary A. K. Pawling J. (2022). Molecular and metabolomic investigation of celecoxib antiproliferative activity in mono-and combination therapy against breast cancer cell models. Anticancer Agents Med. Chem. 22 (8), 1611–1621. 10.2174/1871520621666210910101349 34515014

[B12] Bayraktar S. Baghaki S. Wu J. Liu D. D. Gutierrez-Barrera A. M. Bevers T. B. (2020). Biomarker modulation study of celecoxib for chemoprevention in women at increased risk for breast cancer: a phase II pilot study. Cancer Prev. Res. (Phila) 13 (9), 795–802. 10.1158/1940-6207.CAPR-20-0095 32513785

[B13] Bell C. R. Zelenay S. (2022). COX-2 upregulation by tumour cells post-chemotherapy fuels the immune evasive dark side of cancer inflammation. Cell Stress 6 (9), 76–78. 10.15698/cst2022.09.271 36120509 PMC9442149

[B14] Bell C. R. Pelly V. S. Moeini A. Chiang S. C. Flanagan E. Bromley C. P. (2022). Chemotherapy-induced COX-2 upregulation by cancer cells defines their inflammatory properties and limits the efficacy of chemoimmunotherapy combinations. Nat. Commun. 13 (1), 2063. 10.1038/s41467-022-29606-9 35440553 PMC9018752

[B15] Benelli R. Barboro P. Costa D. Astigiano S. Barbieri O. Capaia M. (2019). Multifocal signal modulation therapy by celecoxib: a strategy for managing castration-resistant prostate cancer. Int. J. Mol. Sci. 20 (23), 6091. 10.3390/ijms20236091 31816863 PMC6929142

[B16] Bi N. Liang J. Zhou Z. Chen D. Fu Z. Yang X. (2019). Effect of concurrent chemoradiation with celecoxib vs concurrent chemoradiation alone on survival among patients with non-small cell lung cancer with and without cyclooxygenase 2 genetic variants: a phase 2 randomized clinical trial. JAMA Netw. Open 2 (12), e1918070. 10.1001/jamanetworkopen.2019.18070 31851351 PMC6991217

[B17] Biase T.M.M.A. Rocha J. G. M. Silva M. T. Ribeiro-Vaz I. Galvão T. F. (2024). Renal effects of selective cyclooxygenase-2 inhibitor anti-inflammatory drugs: a systematic review and meta-analysis. Explor Res. Clin. Soc. Pharm. 15, 100475. 10.1016/j.rcsop.2024.100475 39114538 PMC11304066

[B18] Bray F. Laversanne M. Sung H. Ferlay J. Siegel R. L. Soerjomataram I. (2024). Global cancer statistics 2022: GLOBOCAN estimates of incidence and mortality worldwide for 36 cancers in 185 countries. CA Cancer J. Clin. 74 (3), 229–263. 10.3322/caac.21834 38572751

[B19] Bresalier R. S. Sandler R. S. Quan H. Bolognese J. A. Oxenius B. Horgan K. Adenomatous Polyp Prevention on Vioxx (APPROVe) Trial Investigators (2005). Cardiovascular events associated with rofecoxib in a colorectal adenoma chemoprevention trial. N. Engl. J. Med. 352 (11), 1092–1102. 10.1056/NEJMoa050493 15713943

[B20] Cao Y. Li J. Liang Q. Yang J. Zhang X. Zhang J. (2023). Tumor microenvironment sequential drug/gene delivery nanosystem for realizing multistage boosting of cancer-immunity cycle on cancer immunotherapy. ACS Appl. Mater Interfaces 15 (47), 54898–54914. 10.1021/acsami.3c11394 37963093

[B21] Carpenter P. S. Shepherd H. M. McCrary H. Torrecillas V. Kull A. Hunt J. P. (2018). Association of celecoxib use with decreased opioid requirements after head and neck cancer surgery with free tissue reconstruction. JAMA Otolaryngol. Head. Neck Surg. 144 (11), 988–994. 10.1001/jamaoto.2018.0284 29710229 PMC11849744

[B22] Cecil D. L. Gad E. A. Corulli L. R. Drovetto N. Lubet R. A. Disis M. L. (2022). COX-2 inhibitors decrease expression of PD-L1 in Colon tumors and increase the influx of type I tumor-infiltrating lymphocytes. Cancer Prev. Res. (Phila) 15 (4), 225–231. 10.1158/1940-6207.CAPR-21-0227 34987061 PMC8983455

[B23] Cheki M. Yahyapour R. Farhood B. Rezaeyan A. Shabeeb D. Amini P. (2018). COX-2 in radiotherapy: a potential target for radioprotection and radiosensitization. Curr. Mol. Pharmacol. 11 (3), 173–183. 10.2174/1874467211666180219102520 29468988

[B24] Chiang K. H. Shieh J. M. Shen C. J. Chang T. W. Wu P. T. Hsu J. Y. (2020). Epidermal growth factor-induced COX-2 regulates metastasis of head and neck squamous cell carcinoma through upregulation of angiopoietin-like 4. Cancer Sci. 111 (6), 2004–2015. 10.1111/cas.14400 32227417 PMC7293094

[B25] Ching M. M. Reader J. Fulton A. M. (2020). Eicosanoids in cancer: Prostaglandin E_2_ receptor 4 in cancer therapeutics and immunotherapy. Front. Pharmacol. 11, 819. 10.3389/fphar.2020.00819 32547404 PMC7273839

[B26] Coombes R. C. Tovey H. Kilburn L. Mansi J. Palmieri C. Bartlett J. Randomized European Celecoxib Trial (REACT) Trial Management Group and Investigators (2021). Effect of celecoxib vs placebo as adjuvant therapy on disease-free survival among patients with breast cancer: the REACT randomized clinical trial. JAMA Oncol. 7 (9), 1291–1301. 10.1001/jamaoncol.2021.2193 34264305 PMC8283666

[B27] Dai H. Zhang S. Ma R. Pan L. (2019). Celecoxib inhibits hepatocellular carcinoma cell growth and migration by targeting PNO1. Med. Sci. Monit. 25, 7351–7360. 10.12659/MSM.919218 31568401 PMC6784684

[B28] Daugherty S. E. Pfeiffer R. M. Sigurdson A. J. Hayes R. B. Leitzmann M. Schatzkin A. (2011). Nonsteroidal antiinflammatory drugs and bladder cancer: a pooled analysis. Am. J. Epidemiol. 173 (7), 721–730. 10.1093/aje/kwq437 21367875 PMC3105281

[B29] Davies N. M. McLachlan A. J. Day R. O. Williams K. M. (2000). Clinical pharmacokinetics and pharmacodynamics of celecoxib: a selective cyclo-oxygenase-2 inhibitor. Clin. Pharmacokinet. 38 (3), 225–242. 10.2165/00003088-200038030-00003 10749518

[B30] Dean L. Kane M. (2016). “Celecoxib therapy and *CYP2C9* genotype,” in Medical genetics summaries. Editors Pratt V. M. Scott S. A. Pirmohamed M. Esquivel B. Kattman B. L. Malheiro A. J. (Bethesda (MD): National Center for Biotechnology Information US). 28520369

[B31] Ding Q. Wang Y. Zhang P. Mei L. (2024). Breakthrough in cancer therapy: lutetium texaphyrin-celecoxib conjugate for immune and photodynamic treatment. J. Mater Chem. B 12 (47), 12136–12138. 10.1039/d4tb02019g 39503504

[B32] Dovizio M. Tacconelli S. Ricciotti E. Bruno A. Maier T. J. Anzellotti P. (2012). Effects of celecoxib on prostanoid biosynthesis and circulating angiogenesis proteins in familial adenomatous polyposis. J. Pharmacol. Exp. Ther. 341 (1), 242–250. 10.1124/jpet.111.190785 22262921 PMC3310693

[B33] Edelman M. J. Wang X. Hodgson L. Cheney R. T. Baggstrom M. Q. Thomas S. P. (2017). Phase III randomized, placebo-controlled, double-blind trial of celecoxib in addition to standard chemotherapy for advanced non-small-cell lung cancer with cyclooxygenase-2 overexpression: CALGB 30801 (Alliance). J. Clin. Oncol. 35 (19), 2184–2192. 10.1200/JCO.2016.71.3743 28489511 PMC5493050

[B34] Egashira I. Takahashi-Yanaga F. Nishida R. Arioka M. Igawa K. Tomooka K. (2017). Celecoxib and 2,5-dimethylcelecoxib inhibit intestinal cancer growth by suppressing the Wnt/β-catenin signaling pathway. Cancer Sci. 108 (1), 108–115. 10.1111/cas.13106 27761963 PMC5276826

[B35] Ferrandina G. Ranelletti F. O. Legge F. Salutari V. Martinelli E. Fattorossi A. (2006). Celecoxib up-regulates the expression of the zeta chain of T cell receptor complex in tumor-infiltrating lymphocytes in human cervical cancer. Clin. Cancer Res. 12 (7 Pt 1), 2055–2060. 10.1158/1078-0432.CCR-05-2530 16609015

[B36] Fontana A. Galli L. Fioravanti A. Orlandi P. Galli C. Landi L. (2009). Clinical and pharmacodynamic evaluation of metronomic cyclophosphamide, celecoxib, and dexamethasone in advanced hormone-refractory prostate cancer. Clin. Cancer Res. 15 (15), 4954–4962. 10.1158/1078-0432.CCR-08-3317 19622584

[B37] Gallicchio M. Rosa A. C. Dianzani C. Brucato L. Benetti E. Collino M. (2008). Celecoxib decreases expression of the adhesion molecules ICAM-1 and VCAM-1 in a colon cancer cell line (HT29). Br. J. Pharmacol. 153 (5), 870–878. 10.1038/sj.bjp.0707634 18084318 PMC2267284

[B38] Gandhi S. Slomba R. T. Janes C. Fitzpatrick V. Miller J. Attwood K. (2024). Systemic chemokine-modulatory regimen combined with neoadjuvant chemotherapy in patients with triple-negative breast cancer. J. Immunother. Cancer 12 (11), e010058. 10.1136/jitc-2024-010058 39542655 PMC11575314

[B39] Ghasemi A. Danesh B. Yazdani-Charati J. Hosseinimehr S. J. (2018). Randomized double-blind placebo-controlled trial of celecoxib for the prevention of skin toxicity in patients receiving radiation therapy for breast cancer. Antiinflamm. Antiallergy Agents Med. Chem. 17 (1), 57–67. 10.2174/1871523017666180411162114 29651970

[B40] Greten F. R. Grivennikov S. I. (2019). Inflammation and cancer: triggers, mechanisms, and consequences. Immunity 51 (1), 27–41. 10.1016/j.immuni.2019.06.025 31315034 PMC6831096

[B41] Hamy A. S. Tury S. Wang X. Gao J. Pierga J. Y. Giacchetti S. (2019). Celecoxib with neoadjuvant chemotherapy for breast cancer might worsen outcomes differentially by COX-2 expression and ER status: exploratory analysis of the REMAGUS02 trial. J. Clin. Oncol. 37 (8), 624–635. 10.1200/JCO.18.00636 30702971 PMC6804843

[B42] Hashemi V. Baradaran B. Naseri B. Masoumi J. Baghbani E. Alizadeh N. (2025). The effect of immunomodulatory celecoxsib on the gene expression of inhibitory receptors in dendritic cells generated from monocyte cells. BMC Res. Notes 18 (1), 164. 10.1186/s13104-025-07226-y 40223111 PMC11995585

[B43] Hashemi Goradel N. Najafi M. Salehi E. Farhood B. Mortezaee K. (2019). Cyclooxygenase-2 in cancer: a review. J. Cell Physiol. 234 (5), 5683–5699. 10.1002/jcp.27411 30341914

[B44] Hedayat M. Khezri M. R. Jafari R. Malekinejad H. Majidi Zolbanin N. (2023). Concomitant effects of paclitaxel and celecoxib on genes involved in apoptosis of triple-negative metastatic breast cancer cells. Med. Oncol. 40 (9), 263. 10.1007/s12032-023-02119-1 37548777

[B45] Holt D. M. Ma X. Kundu N. Collin P. D. Fulton A. M. (2012). Modulation of host natural killer cell functions in breast cancer *via* prostaglandin E2 receptors EP2 and EP4. J. Immunother. 35 (2), 179–188. 10.1097/CJI.0b013e318247a5e9 22306906 PMC3721982

[B46] Hou J. Lin Y. Fang Y. Li X. Li X. N. Yang Y. (2021). Clinical efficacy evaluation and prevention of adverse reactions in a randomized trial of a combination of three drugs in the treatment of cancerous pudendal neuralgia. Ann. Palliat. Med. 10 (5), 5754–5762. 10.21037/apm-21-590 33977736

[B47] Hsu A. L. Ching T. T. Wang D. S. Song X. Rangnekar V. M. Chen C. S. (2000). The cyclooxygenase-2 inhibitor celecoxib induces apoptosis by blocking Akt activation in human prostate cancer cells independently of Bcl-2. J. Biol. Chem. 275 (15), 11397–11403. 10.1074/jbc.275.15.11397 10753955

[B48] Hu H. Kang L. Zhang J. Wu Z. Wang H. Huang M. (2022). Neoadjuvant PD-1 blockade with toripalimab, with or without celecoxib, in mismatch repair-deficient or microsatellite instability-high, locally advanced, colorectal cancer (PICC): a single-centre, parallel-group, non-comparative, randomised, phase 2 trial. Lancet Gastroenterol. Hepatol. 7 (1), 38–48. 10.1016/S2468-1253(21)00348-4 34688374

[B49] Hu T. Liu C. J. Yin X. Tang W. Yin L. Bai H. (2023). Selective COX-2 inhibitors do not increase gastrointestinal reactions after colorectal cancer surgery: a systematic review and meta-analysis. BMC Gastroenterol. 23 (1), 281. 10.1186/s12876-023-02918-w 37580670 PMC10426080

[B50] Jahani V. Yazdani M. Badiee A. Jaafari M. R. Arabi L. (2023). Liposomal celecoxib combined with dendritic cell therapy enhances antitumor efficacy in melanoma. J. Control Release 354, 453–464. 10.1016/j.jconrel.2023.01.034 36649743

[B51] Jendrossek V. (2013). Targeting apoptosis pathways by celecoxib in cancer. Cancer Lett. 332 (2), 313–324. 10.1016/j.canlet.2011.01.012 21345578

[B52] Jin K. Qian C. Lin J. Liu B. (2023). Cyclooxygenase-2-Prostaglandin E2 pathway: a key player in tumor-associated immune cells. Front. Oncol. 13, 1099811. 10.3389/fonc.2023.1099811 36776289 PMC9911818

[B53] Kamali K. Nikbakht J. Ayubi E. Nabizadeh M. Sarhadi S. (2020). Comparison of the efficacy of oxybutynin, phenazopyridine, celecoxib, and placebo in the treatment of urinary tract symptoms after BCG therapy in patients with bladder tumors. Urol. J. 18 (4), 439–444. 10.22037/uj.v16i7.5947 32981029

[B54] Kapoor A. Gupta A. Sansar B. Mishra B. K. Gupta P. Singh A. (2025). Triple oral metronomic chemotherapy *versus* chemotherapy of physician discretion after failure of platinum-based therapy in advanced head and neck cancer: a phase III randomized study (METRO-CHASE study). JCO Glob. Oncol. 11, e2500032. 10.1200/GO-25-00032 40403198

[B55] Kim S. H. Kim D. H. Byeon J. Y. Kim Y. H. Kim D. H. Lim H. J. (2017). Effects of CYP2C9 genetic polymorphisms on the pharmacokinetics of celecoxib and its carboxylic acid metabolite. Arch. Pharm. Res. 40 (3), 382–390. 10.1007/s12272-016-0861-2 27864660

[B56] Kim J. Noh M. H. Hur D. Y. Kim B. Kim Y. S. Lee H. K. (2020). Celecoxib upregulates ULBP-1 expression in lung cancer cells *via* the JNK/PI3K signaling pathway and increases susceptibility to natural killer cell cytotoxicity. Oncol. Lett. 20 (6), 279. 10.3892/ol.2020.12142 33014157 PMC7520723

[B57] Kim P. Saba N. F. McCook-Veal A. Liu Y. Klein A. M. Beitler J. J. (2025). Prospective pilot study of second primary tumor prevention with erlotinib and celecoxib in early-stage squamous cell carcinoma of the head and neck: long-term follow-up. Head. Neck 47 (9), 2594–2602. 10.1002/hed.28178 40296503 PMC12339201

[B58] King L. Christie D. Arora D. Anoopkumar-Dukie S. (2020). Cyclooxygenase-2 inhibitors delay relapse and reduce prostate specific antigen (PSA) velocity in patients treated with radiotherapy for nonmetastatic prostate cancer: a pilot study. Prostate Int. 8 (1), 34–40. 10.1016/j.prnil.2019.10.004 32257976 PMC7125379

[B59] Kobayashi K. Kaira K. Kagamu H. (2020). Recovery of the sensitivity to Anti-PD-1 antibody by celecoxib in lung cancer. Anticancer Res. 40 (9), 5309–5311. 10.21873/anticanres.14537 32878822

[B60] Kosaka A. Yajima Y. Yasuda S. Komatsuda H. Nagato T. Oikawa K. (2023). Celecoxib promotes the efficacy of STING-targeted therapy by increasing antitumor CD8^+^ T-cell functions *via* modulating glucose metabolism of CD11b^+^ Ly6G^+^ cells. Int. J. Cancer 152 (8), 1685–1697. 10.1002/ijc.34394 36495276

[B61] Kulp S. K. Yang Y. T. Hung C. C. Chen K. F. Lai J. P. Tseng P. H. (2004). 3-phosphoinositide-dependent protein kinase-1/Akt signaling represents a major cyclooxygenase-2-independent target for celecoxib in prostate cancer cells. Cancer Res. 64 (4), 1444–1451. 10.1158/0008-5472.can-03-2396 14973075

[B62] Landre T. Guetz G. D. Chouahnia K. Fossey-Diaz V. Taleb C. Culine S. (2019). Is there a benefit of addition docetaxel, abiraterone, celecoxib, or zoledronic acid in initial treatments for patients older than 70 years with hormone-sensitive advanced prostate cancer? A meta-analysis. Clin. Genitourin. Cancer. 17 (4), e806–e813. 10.1016/j.clgc.2019.05.001 31227430

[B63] Lemos H. Ou R. McCardle C. Lin Y. Calver J. Minett J. (2020). Overcoming resistance to STING agonist therapy to incite durable protective antitumor immunity. J. Immunother. Cancer 8 (2), e001182. 10.1136/jitc-2020-001182 32847988 PMC7451475

[B64] Li J. Zhu J. MelvinMuscarella W. S. P. Chen C. S. (2006). A structurally optimized celecoxib derivative inhibits human pancreatic cancer cell growth. J. Gastrointest. Surg. 10 (2), 207–214. 10.1016/j.gassur.2005.07.025 16455452

[B65] Li D. Ma Y. Liu W. Ren X. Chen M. Xu X. (2020). Celecoxib combined with salirasib strongly inhibits pancreatic cancer cells in 2D and 3D cultures. Int. J. Med. Sci. 17 (12), 1795–1802. 10.7150/ijms.47546 32714082 PMC7378654

[B66] Li L. Zhang Y. Qin L. (2023). Effect of celecoxib plus standard chemotherapy on cancer prognosis: a systematic review and meta-analysis. Eur. J. Clin. Invest 53 (6), e13973. 10.1111/eci.13973 36807298

[B67] Liao Z. Komaki R. Milas L. Yuan C. Kies M. Chang J. Y. (2005). A phase I clinical trial of thoracic radiotherapy and concurrent celecoxib for patients with unfavorable performance status inoperable/unresectable non-small cell lung cancer. Clin. Cancer Res. 11 (9), 3342–3348. 10.1158/1078-0432.CCR-04-1741 15867233

[B68] Lin J. Z. Hameed I. Xu Z. Yu Y. Ren Z. Y. Zhu J. G. (2018). Efficacy of gefitinib-celecoxib combination therapy in docetaxel-resistant prostate cancer. Oncol. Rep. 40 (4), 2242–2250. 10.3892/or.2018.6595 30066906

[B69] Lipton A. Campbell-Baird C. Witters L. Harvey H. Ali S. (2010). Phase II trial of gemcitabine, irinotecan, and celecoxib in patients with advanced pancreatic cancer. J. Clin. Gastroenterol. 44 (4), 286–288. 10.1097/MCG.0b013e3181cda097 20216081

[B70] Lira M. C. Galluzzi L. Vanpouille-Box C. (2024). COX2-dependent suppression of anticancer immunity. Trends Cancer 10 (7), 573–575. 10.1016/j.trecan.2024.05.006 38821853 PMC11236508

[B71] Liu Z. Xu Y. Liu Z. L. Tian Y. Z. Shen X. H. (2017). Combined application of diclofenac and celecoxib with an opioid yields superior efficacy in metastatic bone cancer pain: a randomized controlled trial. Int. J. Clin. Oncol. 22 (5), 980–985. 10.1007/s10147-017-1133-y 28484877

[B72] Liu Y. He J. Li M. Zhao Z. (2024). Inflammation-driven nanohitchhiker enhances postoperative immunotherapy by alleviating prostaglandin E2-Mediated immunosuppression. ACS Appl. Mater Interfaces 16 (6), 6879–6893. 10.1021/acsami.3c17357 38300288

[B73] Ma H. I. Chiou S. H. Hueng D. Y. Tai L. K. Huang P. I. Kao C. L. (2011). Celecoxib and radioresistant glioblastoma-derived CD133+ cells: improvement in radiotherapeutic effects. Laboratory investigation. J. Neurosurg. 114 (3), 651–662. 10.3171/2009.11.JNS091396 21054139

[B74] Mabrouk A. A. El-Mezayen N. S. Tadros M. I. El-Gazayerly O. N. El-Refaie W. M. (2023). Novel mucoadhesive celecoxib-loaded cubosomal sponges: anticancer potential and regulation of myeloid-derived suppressor cells in oral squamous cell carcinoma. Eur. J. Pharm. Biopharm. 182, 62–80. 10.1016/j.ejpb.2022.12.003 36513316

[B75] Maniewska J. Jeżewska D. (2021). Non-steroidal anti-inflammatory drugs in colorectal cancer chemoprevention. Cancers (Basel) 13 (4), 594. 10.3390/cancers13040594 33546238 PMC7913298

[B76] Mao Y. Sarhan D. Steven A. Seliger B. Kiessling R. Lundqvist A. (2014). Inhibition of tumor-derived prostaglandin-e2 blocks the induction of myeloid-derived suppressor cells and recovers natural killer cell activity. Clin. Cancer Res. 20 (15), 4096–4106. 10.1158/1078-0432.CCR-14-0635 24907113

[B77] Md S. Alhakamy N. A. Alharbi W. S. Ahmad J. Shaik R. A. Ibrahim I. M. (2021). Development and evaluation of repurposed etoricoxib loaded nanoemulsion for improving anticancer activities against lung cancer cells. Int. J. Mol. Sci. 22 (24), 13284. 10.3390/ijms222413284 34948081 PMC8705699

[B78] Meyerhardt J. A. Shi Q. Fuchs C. S. Meyer J. Niedzwiecki D. Zemla T. (2021). Effect of celecoxib vs placebo added to standard adjuvant therapy on disease-free survival among patients with stage III Colon cancer: the CALGB/SWOG 80702 (Alliance) randomized clinical trial. JAMA 325 (13), 1277–1286. 10.1001/jama.2021.2454 33821899 PMC8025124

[B79] Mitryayeva N. Grebinyk L. Artiukh S. Bilozor N. Starenkiy V. (2024). Influence of conformal radiotherapy in combination with radiomodifiers on the content of VEGF, COX-2, and PGE-2 in blood serum of patients with head and neck squamous cell carcinoma. Exp. Oncol. 46 (3), 253–259. 10.15407/exp-oncology.2024.03.253 39704455

[B80] Mohammadi A. Yaghoobi M. M. GholamhoseynianNajar A. Kalantari-Khandani B. Sharifi H. Saravani M. (2016). HSP90 inhibitor enhances anti-proliferative and apoptotic effects of celecoxib on HT-29 colorectal cancer cells *via* increasing BAX/BCL-2 ratio. Cell Mol. Biol. (Noisy-le-grand). 62 (12), 62–67. 10.14715/cmb/2016.62.12.11 27894402

[B81] Mostafa T. M. Alm El-Din M. A. Rashdan A. R. (2022). Celecoxib as an adjuvant to chemotherapy for patients with metastatic colorectal cancer: a randomized controlled clinical study. Saudi Med. J. 43 (1), 37–44. 10.15537/smj.2022.43.1.20210574 35022282 PMC9280566

[B82] Nakai Y. Tanaka N. Asakawa I. Anai S. Miyake M. Morizawa Y. (2020). Biochemical control of the combination of cyclooxygenase-2 inhibitor and 125 I-brachytherapy for prostate cancer: post hoc analysis of an open-label controlled randomized trial. Int. J. Urol. 27 (9), 755–759. 10.1111/iju.14294 32588515

[B83] Negi R. R. Rana S. V. Gupta V. Gupta R. Chadha V. D. Prasad K. K. (2019). Over-expression of Cyclooxygenase-2 in colorectal cancer patients. Asian Pac J. Cancer Prev. 20 (6), 1675–1681. 10.31557/APJCP.2019.20.6.1675 31244287 PMC7021602

[B84] Noronha V. Patil V. M. Menon N. S. Joshi A. Goud S. More S. (2022). Oral metronomic chemotherapy after definitive chemoradiation in esophageal squamous cell carcinoma: a randomized clinical trial. Esophagus 19 (4), 670–682. 10.1007/s10388-022-00923-8 35614161

[B85] O’Rawe M. Wickremesekera A. C. Pandey R. Young D. Sim D. FitzJohn T. (2022). Treatment of glioblastoma with re-purposed renin-angiotensin system modulators: results of a phase I clinical trial. J. Clin. Neurosci. 95, 48–54. 10.1016/j.jocn.2021.11.023 34929651

[B86] Obeid S. Libby P. Husni E. Wang Q. Wisniewski L. M. Davey D. A. (2022). Cardiorenal risk of celecoxib compared with naproxen or ibuprofen in arthritis patients: insights from the PRECISION trial. Eur. Heart J. Cardiovasc Pharmacother. 8 (6), 611–621. 10.1093/ehjcvp/pvac015 35234840

[B87] Ouyang Y. Zhong W. Xu P. Wang B. Zhang L. Yang M. (2024). Tumor-associated neutrophils suppress CD8^+^ T cell immunity in urothelial bladder carcinoma through the COX-2/PGE2/IDO1 axis. Br. J. Cancer 130 (5), 880–891. 10.1038/s41416-023-02552-z 38233491 PMC10912642

[B88] Pak O. Kosianova A. Zaitsev S. Sharma A. Sharma H. Bryukhovetskiy I. (2025). Valproic acid and celecoxib enhance the effect of temozolomide on glioblastoma cells. CNS Neurol. Disord. Drug Targets 24 (5), 375–381. 10.2174/0118715273330268241008220702 39428930

[B89] Pan B. Chen Z. Zhang X. Wang Z. Yao Y. Wu X. (2023). 2,5-dimethylcelecoxib alleviated NK and T-cell exhaustion in hepatocellular carcinoma *via* the gastrointestinal microbiota-AMPK-mTOR axis. J. Immunother. Cancer 11 (6), e006817. 10.1136/jitc-2023-006817 37316264 PMC10277542

[B90] Passaro A. Al Bakir M. Hamilton E. G. Diehn M. André F. Roy-Chowdhuri S. (2024). Cancer biomarkers: emerging trends and clinical implications for personalized treatment. Cell 187 (7), 1617–1635. 10.1016/j.cell.2024.02.041 38552610 PMC7616034

[B91] Patil V. Noronha V. Dhumal S. B. Joshi A. Menon N. Bhattacharjee A. (2020). Low-cost oral metronomic chemotherapy *versus* intravenous cisplatin in patients with recurrent, metastatic, inoperable head and neck carcinoma: an open-label, parallel-group, non-inferiority, randomised, phase 3 trial. Lancet Glob. Health 8 (9), e1213–e1222. 10.1016/S2214-109X(20)30275-8 32827483

[B92] Patil V. M. Noronha V. Menon N. Rai R. Bhattacharjee A. Singh A. (2023). Low-dose immunotherapy in head and neck cancer: a randomized study. J. Clin. Oncol. 41 (2), 222–232. 10.1200/JCO.22.01015 36265101

[B93] Pelly V. S. Moeini A. Roelofsen L. M. Bonavita E. Bell C. R. Hutton C. (2021). Anti-Inflammatory drugs remodel the tumor immune environment to enhance immune checkpoint blockade efficacy. Cancer Discov. 11 (10), 2602–2619. 10.1158/2159-8290.CD-20-1815 34031121 PMC7611767

[B94] Pepine C. J. Gurbel P. A. (2017). Cardiovascular safety of NSAIDs: additional insights after PRECISION and point of view. Clin. Cardiol. 40 (12), 1352–1356. 10.1002/clc.22814 29247518 PMC6490377

[B95] Perroud H. A. Alasino C. M. Rico M. J. Mainetti L. E. Queralt F. Pezzotto S. M. (2016). Metastatic breast cancer patients treated with low-dose metronomic chemotherapy with cyclophosphamide and celecoxib: clinical outcomes and biomarkers of response. Cancer Chemother. Pharmacol. 77 (2), 365–374. 10.1007/s00280-015-2947-9 26721701

[B96] Pritchard R. Rodríguez-Enríquez S. Pacheco-Velázquez S. C. Bortnik V. Moreno-Sánchez R. Ralph S. (2018). Celecoxib inhibits mitochondrial O_2_ consumption, promoting ROS dependent death of murine and human metastatic cancer cells *via* the apoptotic signalling pathway. Biochem. Pharmacol. 154, 318–334. 10.1016/j.bcp.2018.05.013 29800556

[B97] Pu D. Yin L. Huang L. Qin C. Zhou Y. Wu Q. (2021). Cyclooxygenase-2 inhibitor: a potential combination strategy with immunotherapy in cancer. Front. Oncol. 11, 637504. 10.3389/fonc.2021.637504 33718229 PMC7952860

[B98] Qadir A. Khalid Z. Kashan Theba F. Mujtaba Ali M. Asif M. Rizvi F. (2023). Celecoxib and bevacizumab synergistically inhibit non-small cell lung cancer by inducing apoptosis and modulating VEGF and MMP-9 expression. Pak J. Pharm. Sci. 36 (2), 501–506. 10.36721/PJPS.2023.36.2.REG.501-506.1 37530158

[B100] Qian X. Yang H. Ye Z. Gao B. Qian Z. Ding Y. (2024). Celecoxib augments paclitaxel-induced immunogenic cell death in triple-negative breast cancer. ACS Nano 18 (24), 15864–15877. 10.1021/acsnano.4c02947 38829727

[B101] Qin X. Zhang M. Zhao Z. Du Q. Li Q. Jiang Y. (2022). A carrier-free photodynamic nanodrug to enable regulation of dendritic cells for boosting cancer immunotherapy. Acta Biomater. 147, 366–376. 10.1016/j.actbio.2022.05.022 35588995

[B102] Qin H. Li Z. Wu J. Liu X. Wang R. Xu J. (2025). Diclofenac enhances the response of BRAF inhibitor to melanoma through ROS/p38/p53 signaling. Clin. Exp. Pharmacol. Physiol. 52 (3), e70022. 10.1111/1440-1681.70022 39788129

[B103] Qorri B. Harless W. Szewczuk M. R. (2020). Novel molecular mechanism of aspirin and celecoxib targeting mammalian neuraminidase-1 impedes epidermal growth factor receptor signaling axis and induces apoptosis in pancreatic cancer cells. Drug Des. Devel Ther. 14, 4149–4167. 10.2147/DDDT.S264122 33116404 PMC7550724

[B104] Quiñones O. G. Pierre M. B. R. (2019). Cutaneous application of celecoxib for inflammatory and cancer diseases. Curr. Cancer Drug Targets 19 (1), 5–16. 10.2174/1568009618666180430125201 29714143

[B105] Raaijmakers T. K. van den Bijgaart R. J. E. Scheffer G. J. Ansems M. Adema G. J. (2022). NSAIDs affect dendritic cell cytokine production. PLoS One 17 (10), e0275906. 10.1371/journal.pone.0275906 36227963 PMC9560552

[B106] Rao C. V. (2022). Anti-inflammatory drugs decrease the PD-L1 expression and increase the CD8^+^ T-cell infiltration. Cancer Prev. Res. (Phila). 15 (4), 209–211. 10.1158/1940-6207.CAPR-22-0052 35373258

[B107] Rao C. V. Reddy B. S. (2004). NSAIDs and chemoprevention. Curr. Cancer Drug Targets 4 (1), 29–42. 10.2174/1568009043481632 14965265

[B108] Robledo-Cadena D. X. Pacheco-Velázquez S. C. Vargas-Navarro J. L. Padilla-Flores J. A. López-Marure R. Pérez-Torres I. (2024). Synergistic celecoxib and dimethyl-celecoxib combinations block cervix cancer growth through multiple mechanisms. PLoS One 19 (9), e0308233. 10.1371/journal.pone.0308233 39325741 PMC11426494

[B109] Samoudi A. Abolhasani-Zadeh F. Afgar A. Jalilian E. Zeinalynezhad H. Langroudi L. (2024). Treatment of cancer-associated fibroblast-like cells with celecoxib enhances the anti-cancer T helper 1/Treg responses in breast cancer. Naunyn Schmiedeb. Arch. Pharmacol. 398, 6099–6112. 10.1007/s00210-024-03641-3 39652176

[B110] Shayeganmehr D. Ramezannia F. Gharib B. Rezaeilaal A. Shahi F. Jafariazar Z. (2023). Pharmaceutical and clinical studies of celecoxib topical hydrogel for management of chemotherapy-induced hand-foot syndrome. Naunyn Schmiedeb. Arch. Pharmacol. 396 (7), 1571–1581. 10.1007/s00210-022-02339-8 36418469

[B111] Shen C. J. Chen H. C. Lin C. L. Thakur A. Onuku R. Chen I. C. (2025). Contribution of prostaglandin E2-Induced neuronal excitation to drug resistance in glioblastoma countered by a novel blood-brain barrier crossing celecoxib derivative. Adv. Sci. (Weinh) 12, e06336. 10.1002/advs.202506336 40658067 PMC12520548

[B112] Sigler S. Abdel-Halim M. Fathalla R. K. Da Silva L. M. Keeton A. B. Maxuitenko Y. Y. (2025). Novel celecoxib derivative, RF26, blocks Colon cancer cell growth by inhibiting PDE5, activating cGMP/PKG signaling, and suppressing β-catenin-dependent transcription. Anticancer Agents Med. Chem. 25 (1), 52–62. 10.2174/0118715206318802240821114353 39225209

[B113] Singh S. (2018). Liposome encapsulation of doxorubicin and celecoxib in combination inhibits progression of human skin cancer cells. Int. J. Nanomedicine 13 (T-NANO 2014 Abstracts), 11–13. 10.2147/IJN.S124701 29593389 PMC5863640

[B114] Sun J. Liu N. B. Zhuang H. Q. Zhao L. J. Yuan Z. Y. Wang P. (2017). Celecoxib-erlotinib combination treatment enhances radiosensitivity in A549 human lung cancer cell. Cancer Biomark. 19 (1), 45–50. 10.3233/CBM-160323 28282799 PMC13020707

[B115] Sung M. W. Lee D. Y. Park S. W. Oh S. M. Choi J. J. Shin E. S. (2017). Celecoxib enhances the inhibitory effect of 5-FU on human squamous cell carcinoma proliferation by ROS production. Laryngoscope 127 (4), E117–E123. 10.1002/lary.26309 27666139

[B116] Swanton C. Bernard E. Abbosh C. André F. Auwerx J. Balmain A. (2024). Embracing cancer complexity: hallmarks of systemic disease. Cell 187 (7), 1589–1616. 10.1016/j.cell.2024.02.009 38552609 PMC12077170

[B117] Tai Y. Zhang L. H. Gao J. H. Zhao C. Tong H. Ye C. (2019). Suppressing growth and invasion of human hepatocellular carcinoma cells by celecoxib through inhibition of cyclooxygenase-2. Cancer Manag. Res. 11, 2831–2848. 10.2147/CMAR.S183376 31114336 PMC6497485

[B118] Tan T. Fu X. Qu J. Zhang M. Chen H. Wang Y. (2021). 2,5-dimethyl celecoxib induces apoptosis and autophagy *via* activation of ROS/JNK axis in nasopharyngeal carcinoma cells. Aging (Albany NY) 13 (17), 21483–21496. 10.18632/aging.203488 34511433 PMC8457580

[B119] Tang B. Guo Z. S. Bartlett D. L. Yan D. Z. Schane C. P. Thomas D. L. (2020). Synergistic combination of oncolytic virotherapy and immunotherapy for glioma. Clin. Cancer Res. 26 (9), 2216–2230. 10.1158/1078-0432.CCR-18-3626 32019860 PMC7723446

[B120] Thi Thanh Nguyen N. Yoon L. S. (2024). Celecoxib and sulindac sulfide elicit anticancer effects on PIK3CA-mutated head and neck cancer cells through endoplasmic reticulum stress, reactive oxygen species, and mitochondrial dysfunction. Biochem. Pharmacol. 224, 116221. 10.1016/j.bcp.2024.116221 38641308

[B121] Tian J. Guo F. Chen Y. Li Y. Yu B. Li Y. (2019). Nanoliposomal formulation encapsulating celecoxib and genistein inhibiting COX-2 pathway and Glut-1 receptors to prevent prostate cancer cell proliferation. Cancer Lett. 448, 1–10. 10.1016/j.canlet.2019.01.002 30673592

[B122] Tołoczko-Iwaniuk N. Dziemiańczyk-Pakieła D. Nowaszewska B. K. Celińska-Janowicz K. Miltyk W. (2019). Celecoxib in cancer therapy and prevention - review. Curr. Drug Targets 20 (3), 302–315. 10.2174/1389450119666180803121737 30073924

[B123] Trifan O. C. Hla T. (2003). Cyclooxygenase-2 modulates cellular growth and promotes tumorigenesis. J. Cell Mol. Med. 7 (3), 207–222. 10.1111/j.1582-4934.2003.tb00222.x 14594546 PMC6741314

[B124] Tseng P. H. Wang Y. C. Weng S. C. Weng J. R. Chen C. S. Brueggemeier R. W. (2006). Overcoming trastuzumab resistance in HER2-overexpressing breast cancer cells by using a novel celecoxib-derived phosphoinositide-dependent kinase-1 inhibitor. Mol. Pharmacol. 70 (5), 1534–1541. 10.1124/mol.106.023911 16887935

[B125] Tudor D. V. Bâldea I. Olteanu D. E. Fischer-Fodor E. Piroska V. Lupu M. (2021). Celecoxib as a valuable adjuvant in cutaneous melanoma treated with trametinib. Int. J. Mol. Sci. 22 (9), 4387. 10.3390/ijms22094387 33922284 PMC8122835

[B126] Valverde A. Peñarando J. Cañas A. López-Sánchez L. M. Conde F. Guil-Luna S. (2017). The addition of celecoxib improves the antitumor effect of cetuximab in colorectal cancer: role of EGFR-RAS-FOXM1-β- catenin signaling axis. Oncotarget 8 (13), 21754–21769. 10.18632/oncotarget.15567 28423516 PMC5400621

[B127] Veltman J. D. Lambers M. E. van Nimwegen M. Hendriks R. W. Hoogsteden H. C. Aerts J. G. J. V. (2010). COX-2 inhibition improves immunotherapy and is associated with decreased numbers of myeloid-derived suppressor cells in mesothelioma. Celecoxib influences MDSC function. BMC Cancer 10, 464. 10.1186/1471-2407-10-464 20804550 PMC2939552

[B128] Wang D. Dubois R. N. (2010). Eicosanoids and cancer. Nat. Rev. Cancer 10 (3), 181–193. 10.1038/nrc2809 20168319 PMC2898136

[B129] Wang L. W. Hsiao C. F. Chen W. T. Lee H. H. Lin T. C. Chen H. C. (2014). Celecoxib plus chemoradiotherapy for locally advanced rectal cancer: a phase II TCOG study. J. Surg. Oncol. 109 (6), 580–585. 10.1002/jso.23538 24374744

[B130] Wang G. Li J. Zhang L. Huang S. Zhao X. Zhao X. (2017). Celecoxib induced apoptosis against different breast cancer cell lines by down-regulated NF-κB pathway. Biochem. Biophys. Res. Commun. 490 (3), 969–976. 10.1016/j.bbrc.2017.06.148 28666869

[B131] Wen B. Wei Y. T. Mu L. L. Wen G. R. Zhao K. (2020). The molecular mechanisms of celecoxib in tumor development. Med. Baltim. 99 (40), e22544. 10.1097/MD.0000000000022544 33019464 PMC7535670

[B132] Xiao J. Wang F. Lu H. Xu S. Zou L. Tian Q. (2019). Targeting the COX2/MET/TOPK signaling axis induces apoptosis in gefitinib-resistant NSCLC cells. Cell Death Dis. 10 (10), 777. 10.1038/s41419-019-2020-4 31611604 PMC6791885

[B133] Xie L. Li R. Zheng B. Xie Z. Fang X. Dai T. (2021). One-step transformation from rofecoxib to a COX-2 NIR probe for human cancer tissue/organoid targeted bioimaging. ACS Appl. Bio Mater 4 (3), 2723–2731. 10.1021/acsabm.0c01634 35014311

[B134] Xu H. B. Shen F. M. Lv Q. Z. (2015). Celecoxib enhanced the cytotoxic effect of cisplatin in drug-resistant human gastric cancer cells by inhibition of cyclooxygenase-2. Eur. J. Pharmacol. 769, 1–7. 10.1016/j.ejphar.2015.09.025 26407653

[B135] Xu H. B. Shen F. M. Lv Q. Z. (2016). Celecoxib enhanced the cytotoxic effect of cisplatin in chemo-resistant gastric cancer xenograft mouse models through a cyclooxygenase-2-dependent manner. Eur. J. Pharmacol. 776, 1–8. 10.1016/j.ejphar.2016.02.035 26879869

[B136] Xu H. Li P. Ma H. Tan Y. Wang X. Cai F. (2023). ADT-OH synergistically enhanced the antitumor activity of celecoxib in human colorectal cancer cells. Cancer Med. 12 (16), 17193–17211. 10.1002/cam4.6342 37492969 PMC10501245

[B137] Xue W. P. Bai S. M. Luo M. Bi Z. F. Liu Y. M. Wu S. K. (2011). Phase I clinical trial of nasopharyngeal radiotherapy and concurrent celecoxib for patients with locoregionally advanced nasopharyngeal carcinoma. Oral Oncol. 47 (8), 753–757. 10.1016/j.oraloncology.2011.06.002 21708478

[B138] Xun X. Zhang C. Wang S. Hu S. Xiang X. Cheng Q. (2021). Cyclooxygenase-2 expressed hepatocellular carcinoma induces cytotoxic T lymphocytes exhaustion through M2 macrophage polarization. Am. J. Transl. Res. 13 (5), 4360–4375. 34150019 PMC8205841

[B139] Ye S. Y. Li J. Y. Li T. H. Song Y. X. Sun J. X. Chen X. W. (2022). The efficacy and safety of celecoxib in addition to standard cancer therapy: a systematic review and meta-analysis of randomized controlled trials. Curr. Oncol. 29 (9), 6137–6153. 10.3390/curroncol29090482 36135051 PMC9497539

[B140] Yeomans N. D. Graham D. Y. Husni M. E. Solomon D. H. Stevens T. Vargo J. (2018). Randomised clinical trial: gastrointestinal events in arthritis patients treated with celecoxib, ibuprofen or naproxen in the PRECISION trial. Aliment. Pharmacol. Ther. 47 (11), 1453–1463. 10.1111/apt.14610 29667211

[B141] Yilmaz Ç. Köksoy S. Çeker T. Aslan M. (2021). Diclofenac down-regulates COX-2 induced expression of CD44 and ICAM-1 in human HT29 colorectal cancer cells. Naunyn Schmiedeb. Arch. Pharmacol. 394 (11), 2259–2272. 10.1007/s00210-021-02139-6 34436652

[B142] Yin D. Jin G. He H. Zhou W. Fan Z. Gong C. (2021). Celecoxib reverses the glioblastoma chemo-resistance to temozolomide through mitochondrial metabolism. Aging (Albany NY) 13 (17), 21268–21282. 10.18632/aging.203443 34497154 PMC8457578

[B143] Zelenay S. van der Veen A. G. et L. Snelgrove K. J. Rogers N. Acton S. E. (2015). Cyclooxygenase-dependent tumor growth through evasion of immunity. Cell 162 (6), 1257–1270. 10.1016/j.cell.2015.08.015 26343581 PMC4597191

[B144] Zhang H. Tian M. Xiu C. Wang Y. Tang G. (2013). Enhancement of antitumor activity by combination of tumor lysate-pulsed dendritic cells and celecoxib in a rat glioma model. Oncol. Res. 20 (10), 447–455. 10.3727/096504013x13685487925176 24308155

[B145] Zhang P. He D. Song E. Jiang M. Song Y. (2019). Celecoxib enhances the sensitivity of non-small-cell lung cancer cells to radiation-induced apoptosis through downregulation of the Akt/mTOR signaling pathway and COX-2 expression. PLoS One 14 (10), e0223760. 10.1371/journal.pone.0223760 31613929 PMC6793859

[B146] Zhang W. Yi L. Shen J. Zhang H. Luo P. (2020). Comparison of the benefits of celecoxib combined with anticancer therapy in advanced non-small cell lung cancer: a meta-analysis. J. Cancer 11 (7), 1816–1827. 10.7150/jca.35003 32194793 PMC7052875

[B147] Zhang P. Song E. Jiang M. Song Y. (2021). Celecoxib and afatinib synergistic enhance radiotherapy sensitivity on human non-small cell lung cancer A549 cells. Int. J. Radiat. Biol. 97 (2), 170–178. 10.1080/09553002.2021.1846817 33164600

[B148] Zhang M. Qin X. Zhao Z. Du Q. Li Q. Jiang Y. (2022). A self-amplifying nanodrug to manipulate the Janus-faced nature of ferroptosis for tumor therapy. Nanoscale Horiz. 7 (2), 198–210. 10.1039/d1nh00506e 35023537

[B149] Zhang X. Liang Q. Cao Y. Yang T. An M. Liu Z. (2024). Dual depletion of myeloid-derived suppressor cells and tumor cells with self-assembled gemcitabine-celecoxib nano-twin drug for cancer chemoimmunotherapy. J. Nanobiotechnol. 22 (1), 319. 10.1186/s12951-024-02598-y 38849938 PMC11161946

[B150] Zheng L. Qin S. Si W. Wang A. Xing B. Gao R. (2021). Pan-cancer single-cell landscape of tumor-infiltrating T cells. Science 374 (6574), abe6474. 10.1126/science.abe6474 34914499

[B151] Zhu J. May S. Ulrich C. Stockfleth E. Eberle J. (2021). High ROS production by celecoxib and enhanced sensitivity for death ligand-induced apoptosis in cutaneous SCC cell lines. Int. J. Mol. Sci. 22 (7), 3622. 10.3390/ijms22073622 33807213 PMC8036359

[B152] Zhu X. Zhang W. Yu Z. Yang X. Li L. Chen C. (2024). Synergistic action of gemcitabine and celecoxib in promoting the antitumor efficacy of anti-programmed death-1 monoclonal antibody by triggering immunogenic cell death. Transl. Cancer Res. 13 (6), 3031–3045. 10.21037/tcr-24-698 38988937 PMC11231791

